# CDK7 inhibitor THZ1 inhibits MCL1 synthesis and drives cholangiocarcinoma apoptosis in combination with BCL2/BCL-XL inhibitor ABT-263

**DOI:** 10.1038/s41419-019-1831-7

**Published:** 2019-08-09

**Authors:** Tianlu Huang, Xiwei Ding, Guifang Xu, Gang Chen, Yu Cao, Chunyan Peng, Shanshan Shen, Ying Lv, Lei Wang, Xiaoping Zou

**Affiliations:** 1 Department of Gastroenterology, Nanjing Drum Tower Hospital, The Affiliated Hospital of Nanjing University Medical School, Nanjing, 210008 Jiangsu China; 2 Division of Hepatobiliary Surgery, The First Affiliated Hospital of Wenzhou Medical University, Wenzhou, 325000 Zhejiang China

**Keywords:** Bile duct cancer, Bile duct cancer

## Abstract

Cholangiocarcinoma (CCA) is a fatal disease without effective targeted therapy. We screened a small-molecule library of 116 inhibitors targeting different targets of the cell cycle and discovered several kinases, including Cyclin-dependent kinase 7 (CDK7) as vulnerabilities in CCA. Analysis of multiple CCA data sets demonstrated that CDK7 was overexpressed in CCA tissues. Further studies demonstrated that CDK7 inhibitor THZ1 inhibited cell viability and induced apoptosis in CCA cells. We also showed that THZ1 inhibited CCA cell growth in a xenograft model. RNA-sequencing followed by Gene ontology analysis showed a striking impact of THZ1 on DNA-templated transcriptional programs. THZ1 downregulated CDK7-mediated phosphorylation of RNA polymerase II, indicative of transcriptional inhibition. A number of oncogenic transcription factors and survival proteins, like MCL1, FOSL1, and RUNX1, were repressed by THZ1. MCL1, one of the antiapoptotic BCL2 family members, was significantly inhibited upon THZ1 treatment. Accordingly, combining THZ1 with a BCL2/BCL-XL inhibitor ABT-263 synergized in impairing cell growth and driving apoptosis. Our results demonstrate CDK7 as a potential target in treating CCA. Combinations of CDK7 inhibition and BCL2/BCL-XL inhibition may offer a novel therapeutic strategy for CCA.

## Introduction

Cholangiocarcinoma (CCA) is a malignancy with an upward trend in the incidence and mortality all over the world. Lack of apparent clinical presentation or biomarkers for diagnosis at early stages, most patients are not eligible for surgical resection or liver transplantation while exhibiting symptoms^1^. Also, CCA shows poor response to chemotherapy, and no effective targeted therapeutics has been approved. In the past several decades, the 5-year survival rate for CCA has not improved, which is still around 10%. As a result, it is necessary to find therapeutic agents and develop innovative strategies for improving patients’ outcome.

CCA is a complex disease and the pathogenesis is still unclear. In 2016, Yang et al. identified 48 differentially expressed transcription factors in CCA compared with normal tissues by conducting integrated analysis of multiple CCA microarray data^2^. They also found that the cell cycle was the most significant enriched pathway in CCA. The results of this research indicate that inhibitors targeting cell cycle-related genes may be effective in CCA treatment. In this study, we used a library containing 116 small inhibitors, targeting different components in the cell cycle pathway, to perform an unbiased screening. As a result, we observed several kinases as vulnerabilities in CCA. Among them, we focused on Cyclin-dependent kinase (CDK) 7 and demonstrated CDK7 inhibition as a potent therapeutic approach.

CDK7 is a member of the CDK family, which plays an important role in cell cycle and transcription regulation. It activates other CDKs, including CDK1, CDK2, CDK4, and CDK6, by forming a cyclin-activating kinase (CAK) with cyclin H and MAT1. Besides cell cycle progression, CDK7 also affects transcriptional activity by phosphorylating the C-terminal domain of RNA polymerase II (RNP2)^3,4^. It has also been reported that CDK7 is associated with super-enhancers, which play important roles in cancer cell death^5^. In recent years, several researchers demonstrated that CDK7 was overexpressed in different cancers and correlated with poor prognosis^6–8^. CDK7 has become a potential therapeutic target, and CDK7 inhibitors have entered the clinical trial as promising methods for a variety of malignancies. THZ1 is a covalent inhibitor of CDK7, which shows strong antitumor effects in various cancers by inhibition of CDK7^9–16^. However, the role of CDK7 and the effect of THZ1 treatment in CCA have not been reported. Here, our research aims to reveal therapeutic potential of CDK7 inhibition in CCA preclinical models and further elucidate the possibility of combining THZ1 with other inhibitors to enhance antitumor effect in CCA treatment.

## Materials and methods

### Cell lines and cell culture

Human CCA cell lines HuCCT1, HuH28, and OZ were kindly provided by Lewis R. Roberts (Mayo Clinic, MN, USA), which were originally obtained from the Japanese Collection of Research Bioresources. Human CCA cell lines RBE and HCCC9810 were obtained from Shanghai Cell Bank of Chinese Academy of Sciences (Shanghai, China). All CCA cell lines were authenticated using short-tandem repeat profiling. All cell lines used were maintained in RPMI 1640 (Gibco, Grand Island, NY, USA) supplemented with 10% fetal calf serum (Biological Industries, Kibbutz Beit Haemek, Israel) and cultured at 37% in the presence of 5% CO_2_.

### Inhibitors and antibodies

DiscoveryProbe^TM^ Cell Cycle Compound Library (L1037) was purchased from APExBIO (Houston, TX, USA). THZ1, THZ1-R, ABT-263 (Navitoclax), Triptolide, and ICEC0942 were purchased from Medchem Express (Monmouth Junction, NJ, USA). Inhibitors were dissolved in Dimethyl sulfoxide (DMSO) at a stock concentration and stored at −80 °C until used. Antibodies used for western blotting were as follows: Anti-β-actin primary antibody was purchased from Sigma Aldrich (St. Louis, MO, USA). Antibodies against human cleaved PARP (#5625), BCL-XL (#2764), MCL1 (#5453), and CDK7 (#2916) were purchased from Cell Signaling Technology (Beverly, MA, USA). Antibodies against human RNP2 (A300-653A-T), RNP2-p-S2 (A300-654A-T), and RNP2-p-S5 (A304-408A-T) were from Bethyl Laboratories (Montgomery, TX, USA). An antibody against human RNP2-p-S7 (04-1570) was purchased from Millipore (Burlington, MA, USA). Antibody against human BCL2 (SC-7382) was from Santa Cruz (Santa Cruz, CA, USA).

### RNA extraction and cDNA synthesis

After designated treatments, total RNA was extracted from cells using RNeasy Plus Mini Kit from QIAGEN (Duesseldorf, Germany) according to the instruction. cDNA was synthesized from 1000 ng mRNA using High Capacity cDNA Reverse Transcription Kits (TaKaRA, Tokyo, Japan) following the manufacturer’s protocol. Reverse transcription was carried using a DNA thermal cycler (Bio-Rad, Hercules, CA, USA). The thermal conditions were 37 °C for 15 min and 85 °C for 5 s, and cDNA was stored at 4 °C.

### Real-time qPCR and relative quantification of mRNA expression

Real-time qPCR was performed using the SYBR qPCR detection Kit (TaKaRA, Tokyo, Japan) and the Real-time PCR System (Roche, Basel, Switzerland). PCR was performed in the relative quantification of mRNA expression using the comparative cycle threshold (C_T_) method. 18S expression was used as the endogenous control. The primers used were listed in Supplementary Table 1.

### Western blotting analysis

Whole-cell lysates were extracted using cell extraction buffer (Invitrogen, Carlsbad, CA, USA). Proteins were resolved by SDS-PAGE and transferred to polyvinylidene difluoride membranes. Membranes were blotted with primary antibodies at the following dilutions: cleaved PARP (1:1000), BCL-XL (1:1000), MCL1 (1:1000), CDK7 (1:1000), RNP2 (1:1000), RNP2-p-S2 (1:1000), RNP2-p-S5 (1:1000), RNP2-p-S7 (1:1000), BCL2 (1:200), and β-actin (1:5000). Membranes were exposed to Chemiluminescenthorseradish peroxidase detection kit (Millipore, Burlington, MA, USA) and signals were visualized using 5200 Multi Chemiluminescent Imaging System (Tanon, China).

### Immunohistochemistry

Immunohistochemistry (IHC) was performed for CDK7 according to the standard methods. The cholangiocarcinoma tissue microarray slides (HBiDC122Su01) were obtained from SHANGHAI OUTDO BIOTECH (Shanghai, China). The microarray was built using cholangiocarcinoma and nontumoral surrounding tissues from National Human Genetic Resources Sharing Service Platform (2005DKA213000). The study methodologies conformed to the standards set by the Declaration of Helsinki. The intensity of CDK7 expression was graded as follows: 0 (negative), 1 (weak), 2 (moderate), and 3 (strong). The percentage of staining was graded as follows: 0 (no positive cells), 1 (<25% positive cells), 2 (25–49% positive cells), 3 (50–74% positive cells), and 4 (≥75% positive cells). The total score was calculated by combining the two parameters. Immunohistochemical staining was analyzed by two pathologists in a blinded manner. IHC Score ≥4 was considered positive staining.

### RNA interfering

Cells were seeded into six-well plates at a density of 1–2 × 10^5^ cells/well and reversely transfected using the siRNA lipid, which was mixed with 5 μl of 20 μM siRNA and 5 μl Lipofectamine RNAiMAX (Life Technologies) in 250 μl of Opti-MEM (Gibco). The siRNAs were synthesized by GenePharma (Shanghai, China) and listed in Supplementary Table 2.

### Cell viability assay

Cell viability was detected by Cell counting kit-8 (CCK-8) kit from Dojindo Laboratories (Kyushu, Japan). 2000–3000 cells were seeded into 96-well clear plates in triplicate, cultured overnight, and then treated with different reagents for indicated time. Ten microliters of the tetrazolium substrate was added to each well. After maintaining at 37 °C for 1–2 h, the absorbance at 450 nm was measured. For analysis of synergy between THZ1 with ABT-263, cells were treated with single drugs or different drug combinations for 48 h and cell viability was determined by the CCK-8 assay. The combination index (CI) was determined using CompuSyn software^17^. CI<1 indicates synergism.

### BrdU cell proliferation ELISA assay

Cells were plated in 96-well plates at 3000 cells/well in triplicate, cultured overnight, and then treated with different reagents for indicated time. Cell proliferation assay was performed using the BrdU cell proliferation ELISA kit from Roche (Basel, Switzerland) according to the manufacturer’s instructions.

### Annexin V-FITC apoptosis assay

Cells were seeded in a six-well plate at 1 × 10^5^ cells/well and treated with different reagents for indicated time after adhering overnight. Apoptosis was assessed by flow cytometry using the Annexin V-FITC Apoptosis Detection kit (BD Pharmingen, Franklin Lakes, NJ, USA) and performed according to the manufacturer’s instruction. Data were analyzed using FlowJo software.

### Caspase3/7 activity assay

Caspase3/7 activity was analyzed using the Caspase-Glo 3/7 assay kit (Promega, Madison, WI, USA) following manufacturer’s instructions. Three thousand cells were seeded into 96-well white opaque plate and a corresponding optically 96-well clear plate and then adhere overnight. Then, cells were treated with different reagents and at the end of the incubation time, Caspase-Glo reagent was added to each well. The luminescence was measured in a GloMax Luminometer (Promega). Caspase 3/7 activity was normalized to the relative viable cell number, which was evaluated by the corresponding 96-well clear plate with the CCK-8 assay.

### RNA sequencing and analysis

HuCCT1, HuH28, and RBE cells were grown in six-well plates, treated with vehicle or THZ1 (25 nM and 200 nM) for 6 h and total RNA was isolated as described above. RNA sequencing service was provided by Genewiz (Suzhou, China).

Briefly, sequencing libraries were prepared using TruSeq Library Prep Kit (Illumina) and were sequenced on HiSeq2500 sequencer (Illumina). Reads were aligned to human hg19 and gene expression values were calculated by counting the reads mapping using HTseqcount46. Expression level was measured as FPKM using stringtie software. *P* values were adjusted using the Benjamini–Hochberg method for controlling the false discovery rate. Genes with an adjusted *P* value < 0.01 and fold change ≥2 were considered differentially expressed.

### Xenograft assays in nude mice

Female nude mice (5-to-6-week-old) were purchased from Beijing Vital River Laboratory Animal Technology. The animal experiments were approved by the Institutional Animal Care and Use Committee of Nanjing Drum Tower Hospital (20181101). HuCCT1 cells (3 × 10^6^ cells) were suspended in 100 μl phosphate buffer solution, mixed with 100 μl Matrigel and injected subcutaneously into the right flank of nude mice. When the tumor size reached about 200 mm^3^, mice were randomly separated into two groups and treated intraperitoneally (i.p.) with either vehicle (10% DMSO and 90% dextrose 5% in water) or THZ1 (10 mg/kg, twice daily) for 27 days. The size of the tumors and the weight of mice were measured every 3–4 days and at the end of treatment, mice were sacrificed. Tumor size was measured with digital caliper and calculated as *V* = *L* × *S*^2^/2 (where *L* is the longest diameter and *S* is the shortest diameter).

### Dataset analysis

Publicly available cholangiocarcinoma datasets, GSE26566 dataset^18^, GSE107943 dataset^19^, GSE32225 dataset^20^, GSE76297 dataset^21^, and GSE32879 dataset^22^, were downloaded from Gene Expression Omnibus (GEO) and used to analyze the mRNA expression of CDK7. Moreover, publicly available data (http://firebrowse.org/) generated by The Cancer Genome Atlas (TCGA) Research Network was used to analyze CDK7 expression in different tumors.

### Statistical analysis

All data from western blotting were representative of at least three independent experiments. Statistics tests were conducted with GraphPad Prism 7.0. The IC_50_ value was calculated using nonlinear regression analysis in Prism 7.0. For comparisons between two groups, parametric Student’s *t* test or nonparametric Mann–Whitney test were used. In experiments involving more than two groups, one-way ANOVA with a Turkey post hoc test was used. Gene ontology analyses were performed with DAVID Bioinformatics Resources^23^. *P* < 0.05 was considered statistically significant.

## Results

### CCA cells are prominent sensitivity to inhibitors of CDK7

**Fig. 1:**
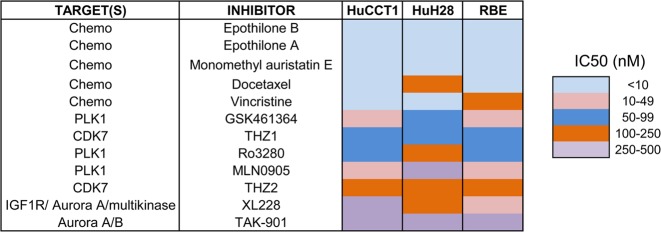
A small-molecule inhibitors library is used to identify potential agents against CCA. Initial screening yielded 34 primary hit compounds that caused over a 50% viability reduction in all three CCA cell lines (HuCCT1, HuH28, and RBE). Second screening yielded 20 primary hit compounds that caused over a 50% viability reduction in all tested CCA cell lines. Subsequent triplicate, dose-dependent analysis was performed to measuring the IC_50_ values of above 20 compounds. Heatmap shows the list of the most effective 12 compounds according to the unbiased small-molecule inhibitors screening

Since cell-cycle was the most significantly enriched pathway in CCA, we carried out an unbiased screening with DiscoveryProbe^TM^ Cell Cycle Compound Library (L1037, APExBIO), containing 116 small-molecule inhibitors. The screening was performed on three CCA cell lines (HuCCT1, HuH28, and RBE). We first treated with all the compounds at the same concentration (1 μM) and 34 of them showed inhibitory rates over 50% (Supplementary Table 3). Next, cells were exposed to these 34 drugs at a second concentration of 0.5 μM. Twenty of them showed inhibitory rates over 50%. Eventually, cells were exposed to these 20 drugs in seven concentrations to determine the IC_50_ value. The cytotoxicity of these inhibitors was ordered by IC_50_ value in three cell lines and top 12 inhibitors were shown in Fig. 1.

Five of these inhibitors were chemotherapeutic drugs. Other compounds were mainly targeting kinases of polo-like kinase 1 (PLK1) and CDK7. Since the role of PLK1 has been studied in CCA before^24^, we then focused on the detailed study exploring the expression and function of CDK7 in CCA and examined the possibility of targeting CDK7 for the treatment of CCA.

### CDK7 is upregulated in human cholangiocarcinoma

**Fig. 2:**
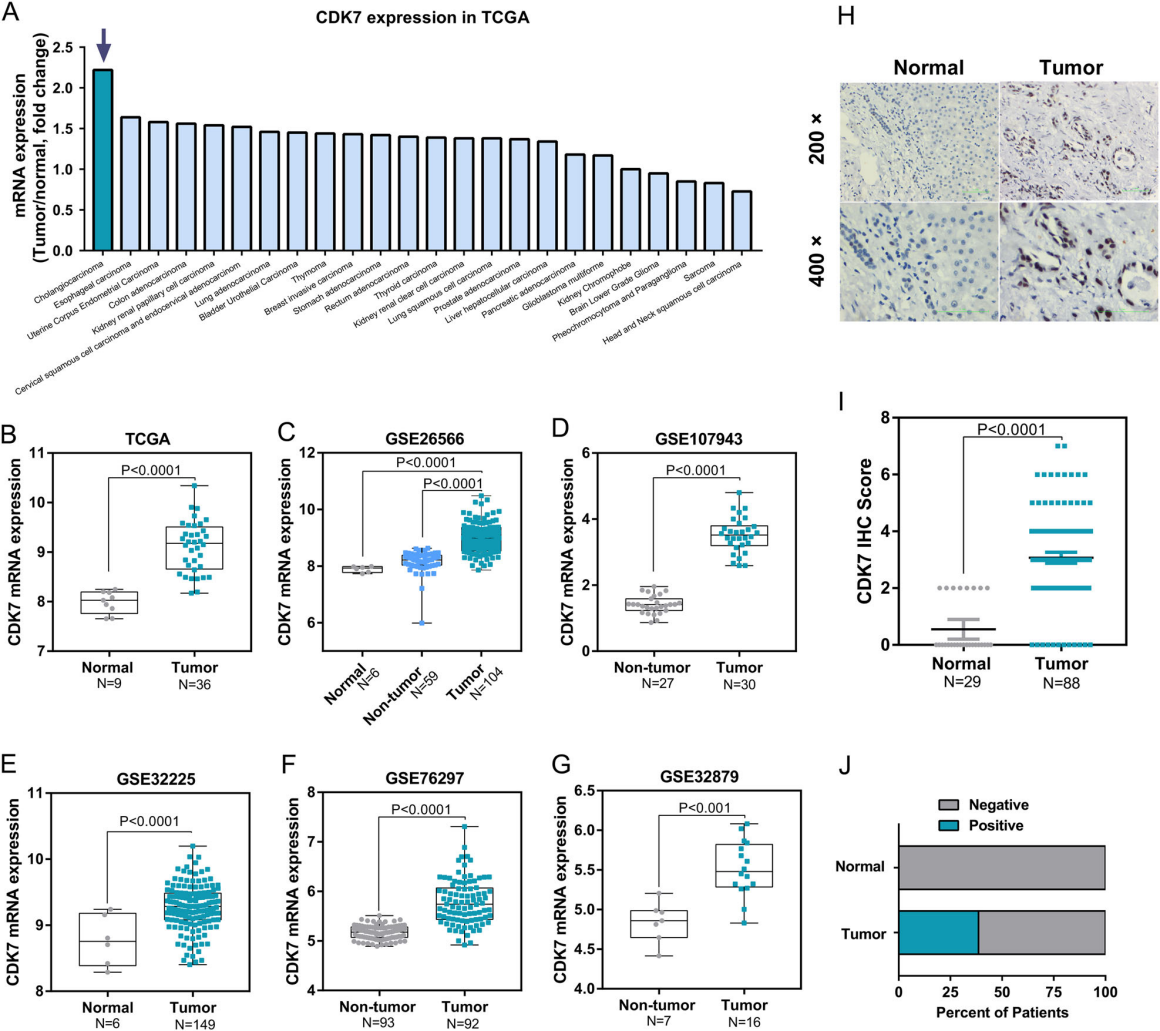
CDK7 expression is upregulated in CCA. **a** CDK7 mRNA expression profile based on tumor/normal fold change in different tumors in TCGA. **b** CDK7 mRNA expression is upregulated in CCA in TCGA. **c**–**g** CDK7 mRNA expression in CCA is higher compared with that in normal biliary tissues or non-tumor liver tissues in GSE26566 dataset, GSE107943 dataset, GSE32225 dataset, GSE76297 dataset and GSE32879 dataset. Data presented were log2 normalized. **h** Immunohistochemical staining for CDK7 performed on 88 human cholangiocarcinoma tissues and 29 adjacent non-tumor tissues. Representative images (magnification, × 200 and 400) were shown. **i** Semi-quantitative analysis of immunohistochemical staining shows that CDK7 protein expression is upregulated in CCA in the tissue microarray. Mann–Whitney test was performed to compare two groups. **j** Bar graph shows the percentage of CDK7 positive expression ratio in tumor and non-tumor tissues in the tissue microarray

To identify the expression of CDK7 in CCA, we analyzed publicly available data from TCGA and GEO datasets. The TCGA database showed that the gene expression of CDK7 was significantly increased in CCA compared with normal bile ducts (Fig. 2a, b). Of note, the elevated CDK7 was found most significant in CCA compared with other cancers in the TCGA database (Fig. 2a). Moreover, CDK7 mRNA expression was significantly higher in CCA than normal bile ducts or surrounding non-tumor liver tissues in GEO GSE26566 dataset (Fig. 2c), GSE107943 dataset (Fig. 2d), GSE32225 dataset (Fig. 2e), GSE76297 dataset (Fig. 2f), and GSE32879 dataset (Fig. 2g). To validate the results from database, we detected protein levels of CDK7 in a tissue microarray of 88 CCA and 29 normal tissues by IHC analysis (Fig. 2h). The CDK7 protein expression was significantly increased in tumor tissues compared with normal bile duct tissues (Fig. 2i). All normal bile ducts in the liver were negative for CDK7, while 38.6% CCA showed positive staining of CDK7 (Fig. 2j). Taken together, CDK7 mRNA and protein levels are significantly upregulated in human CCA, further establishing a therapeutic rationale for targeting CDK7 in CCA.

### THZ1 demonstrates potent antitumor effects against CCA in vitro and in vivo

**Fig. 3:**
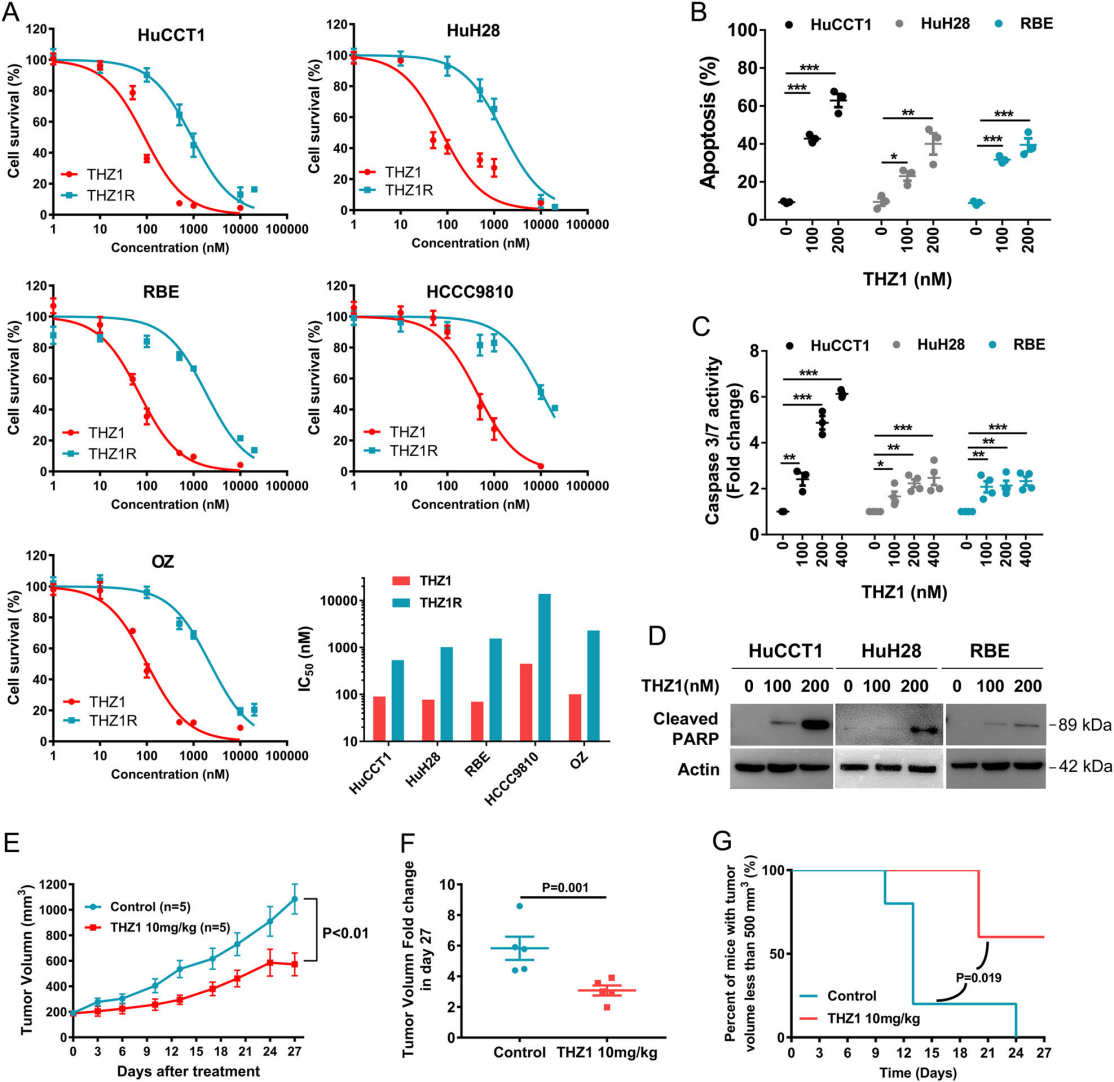
THZ1 exhibits inhibitory potency against CCA in vitro and in vivo. **a** Five CCA cell lines (HuCCT1, HuH28, RBE, HCCC9810, and OZ) were plated in 96-well plates and treated with THZ1 or THZ1R (a THZ1 analog with no significant inhibitory activity of CDK7) at different concentrations for 72 h. Cell viability was measured by CCK-8 assay and dose–response curves show percent cell viability to that of DMSO-treated cells. IC_50_ values were calculated using nonlinear regression analysis in Prism 7.0. Data represent mean ± SEM of three independent replicates. **b** HuCCT1, HuH28, and RBE cells were treated with THZ1 at two concentrations (100 nM and 200 nM) for 72 h and cell apoptosis was measured by Annexin V-FITC/PI staining followed by flow cytometry. Data represent mean ± SEM of three independent replicates. (*P < 0.05; **P < 0.01; ***P < 0.001) **c** HuCCT1, HuH28, and RBE cells were treated with three concentrations (100 nM, 200 nM, and 400 nM) of THZ1 for 48 h for measuring Caspase 3/7 activation. Data represent mean ± SEM of three independent replicates. (*P < 0.05; **P < 0.01; ***P < 0.001) **d** HuCCT1, HuH28, and RBE cells were treated with THZ1 for 24 h and Cleaved PARP was analyzed by western immunoblotting. β-actin served as an internal control. **e** Nude mice were injected subcutaneously with HuCCT1 cells and randomly separated into two groups when tumor size reached around 200 mm^3^. Two groups of mice were treated with THZ1 (10 mg/kg, bid, i.p.) or vehicle (bid, i.p.) for 27 days, separately. Tumor volume was measured twice a week. The growth curve was shown. **f** The fold change of tumor volume at day 27 relative to day 1 was shown as mean ± SEM. **g** The survival curves were assessed by percent of mice with tumor volume <500 mm^3^

To further investigate the effect of CDK7 inhibition on the cell growth of CCA, we treated five CCA cell lines with increasing concentration of THZ1 and measure the cell viability with CCK-8 assay. For each cell line, the IC_50_ value was <500 nM, indicating their high sensitivity to THZ1 (Fig. 3a). We also compared the efficacy of THZ1 with THZ1-R, a THZ1 analog with no pronounced inhibitory activity towards CDK7^10^. The cell growth curves and IC_50_ values demonstrated significant differences between these two compounds (Fig. 3a), suggesting CDK7 could be the vulnerability in CCA cells. BrdU incorporation assay also indicated that THZ1 dramatically inhibited DNA synthesis (Supplementary Fig. 1).

Next, we determined if CDK7 plays a role in the regulation of cell death. Annexin V/FITC assay indicated massive increase in apoptosis rate in THZ1-treated cells compared with non-treated control cells (Fig. 3b and Supplementary Fig. 2). THZ1-induced apoptosis was further evidenced by enhanced Caspase 3/7 activity and poly (ADP-ribose) polymerase (PARP) cleavage (Fig. 3c, d).

We next assessed the antitumor effects of THZ1 in a xenograft tumor model by inoculating HuCCT1 cells subcutaneously into nude mice. Mice bearing tumors were divided into two groups randomly, being treated with vehicle or THZ1 (twice daily, 10 mg/kg), respectively. By recording tumor volume, we discovered that THZ1 significantly suppressed the xenograft growth (Fig. 3e, f and Supplementary Fig. 3). Survival curves (assessed by number of mice with tumor volume <500 mm^3^) showed THZ1 effectively prolonged survival compared with vehicle (Fig. 3g). Of note, THZ1 showed no discernible impact on mice body weight, indicating the bearable toxicity of the treatment (Supplementary Fig. 4).

### THZ1 treatment downregulates global and selective gene transcription in CCA cells

**Fig. 4:**
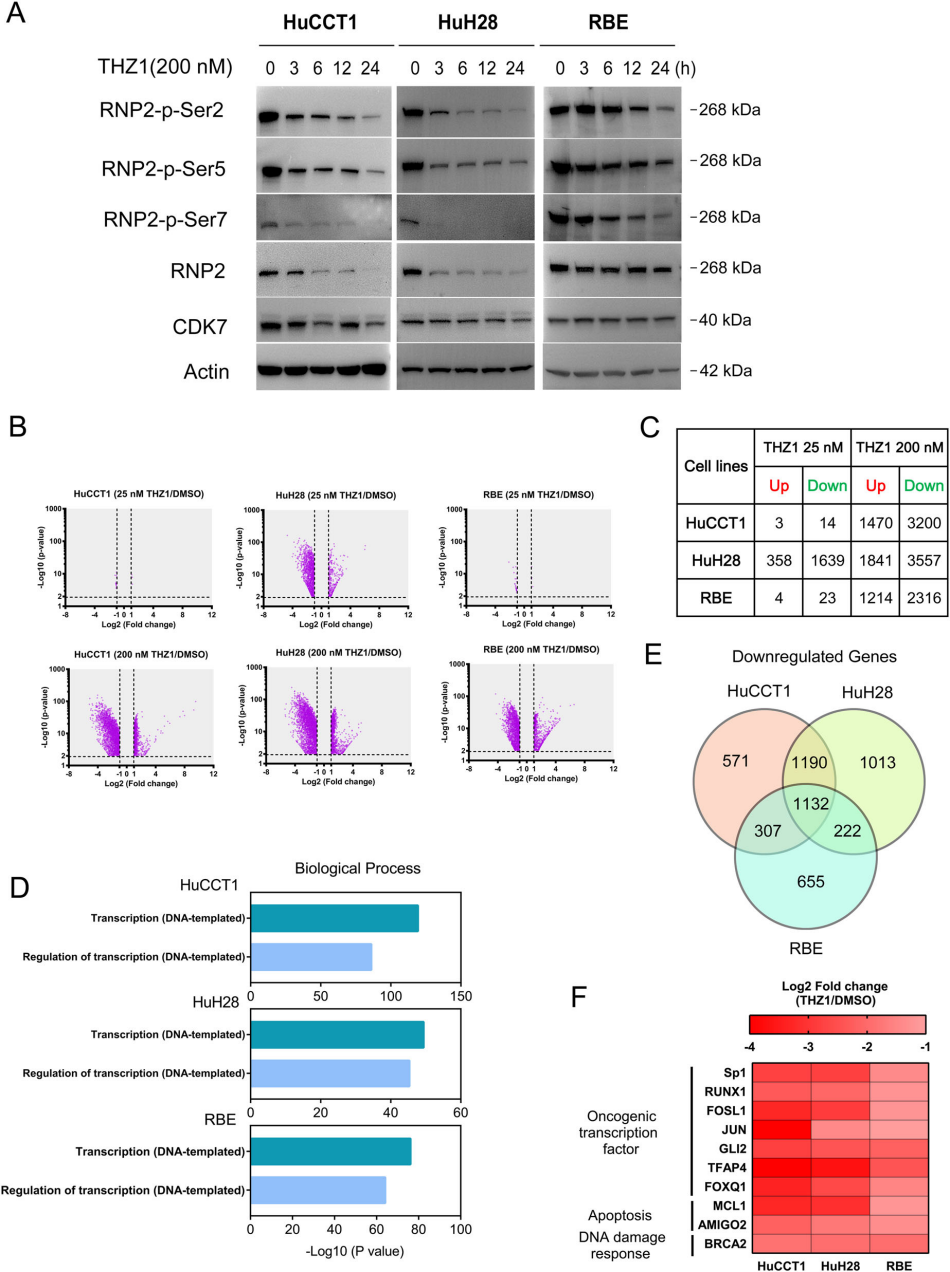
Gene transcription is repressed by THZ1 treatment in CCA cell lines. **a** HuCCT1, RBE, and HuH28 cells were treated with THZ1 at a concentration of 200 nM for 3, 6, 12, and 24 h. RNP2 and RNP2 phosphorylation were analyzed by western blotting. **b** HuCCT1, HuH28, and RBE cells were treated with THZ1 at a concentration of 25 nM or 200 nM for 6 h and then RNA-sequencing was performed. The volcano plots showing the amounts of genes significantly influenced after treatment with THZ1 compared with DMSO. **c** The exact number of genes both significantly up- and downregulated in CCA cell lines exposed to THZ1 is demonstrated (Fold change ≥ 2 with adjusted *P* value < 0.01). **d** Gene Ontology enrichment analysis was performed using significantly downregulated genes in each cell line. **e** The overlaps of genes downregulated in three cell lines are shown in Venn diagram. **f** Heatmap shows the expression levels of some oncogenes (Sp1, RUNX1, FOSL1, JUN, GLI2, TFAP4, FOXQ1, MCL1, AMIGO2, and BRCA2) following treatment in three cell lines

We next aimed to further elucidate the molecular mechanism underlying the impact of THZ1 treatment in CCA cells. Studies have shown that CDK7 regulates transcription by phosphorylating the carboxyl-terminal domain (CTD) of RNA polymerase II (RNP2) at serine 2 (S2), serine 5 (S5), and serine 7 (S7). Along this line, we discovered that THZ1 treatment inhibited RNP2 CTD phosphorylation at S2, S5, and S7 in a time-dependent manner in HuCCT1, HuH28 and RBE cell lines (Fig. 4a). Then, we carried out RNA-sequencing to compare gene expression profile in these three CCA cells following treatment with THZ1 or DMSO. To increase the possibility of disclosing direct effects of THZ1, we exposed cells to THZ1 for only 6 h and then collected RNA samples. Exposure to 200 nM THZ1 for 6 h resulted in a dramatic decrease of global RNA level in all three cell lines (Fig. 4b, c). In contrast, treatment with 25 nM THZ1 for 6 h, which has no or limited inhibitory effect of RNP2-phosphorylation, did not lead to a significant change in RNA expression (Fig. 4b, c). All significantly downregulated or upregulated genes were listed in Supplementary Tables 4–6. Gene Ontology analysis revealed that these THZ1 downregulated transcripts were significantly enriched in processes related to DNA-dependent gene transcription and transcriptional regulation (Fig. 4d and Supplementary Tables 7–9). These transcripts likely mediate the exceptional antitumor property of THZ1.

### THZ1 downregulates antiapoptotic protein MCL1 in CCA

**Fig. 5:**
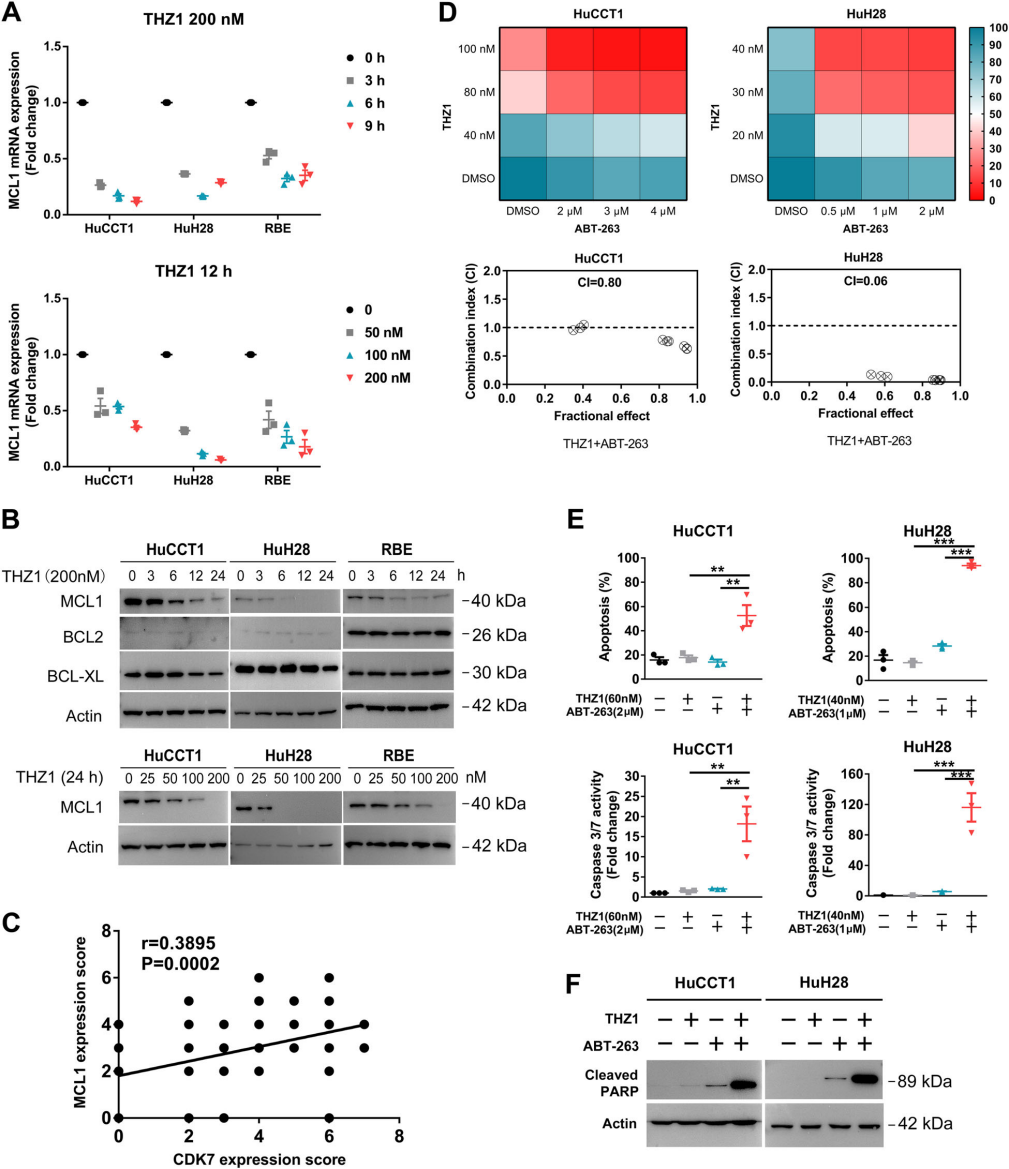
THZ1 represses MCL1 expression and synergizes with ABT-263 in CCA cell lines. **a** Time-dependent effect of THZ1 200 nM and dose-dependent effect of THZ1 12 h on mRNA level of MCL1 were measured by RT-qPCR in indicated cells. Data represent mean ± SEM of three independent replicates. **b** Time-dependent effect of THZ1 200 nM and dose-dependent effect of THZ1 24 h on protein levels of MCL1, BCL2, and BCL-XL were analyzed by western blotting. **c** The expression level of CDK7 showed a significant positive correlation with MCL1 expression in the tissue microarray. The *r* and *P* values were determined by Spearman correlation analysis. **d** The indicated cells were treated with combination of three concentrations of THZ1 and ABT-263 for 48 h. The heatmaps show the results of percent cell viability of three independent results. CI was determined using CompuSyn software. **e** HuCCT1 and HuH28 cells were treated with THZ1 and ABT-263 at indicated concentrations for 48 h. The apoptosis was detected by the Annexin-V/PI assay and Caspase 3/7 activity. Data represent mean ± SEM of three independent replicates (**P < 0.01; ***P < 0.001). **f** HuCCT1 and HuH28 cells were treated with THZ1 and ABT-263 at the above-mentioned concentrations for 48 h. Cleaved PARP protein expression was analyzed by western blotting

Among the genes downregulated after THZ1 treatment, 1132 were overlapped in three cell lines (Fig. 4e), including a number of oncogenes in tumorigenesis like SP1, FOSL1, MCL1, and so on (Fig. 4f). MCL1 is an antiapoptotic member of B cell leukemia-2 (BCL2) family, which consists of pro- and antiapoptotic proteins^25^. A number of studies have revealed MCL1 as a key regulator of survival and apoptosis evasion in CCA cells^26,27^. Real-time qPCR and western blotting validated the results of RNA-Seq. THZ1 downregulated MCL1 mRNA and protein expression in both time- and dose-dependent manner (Fig. 5a, b). Besides MCL1, BCL2, and BCL-XL are the other two important antiapoptotic proteins in the BCL2 family. Interestingly, BCL2 and BCL-XL protein expression did not show apparent decrease following THZ1 treatment (Fig. 5b). We previously showed that MCL1 protein expression was increased in the same CCA tissue microarray^28^. We examined the relevance of CDK7-regulated MCL1 pathway in human subjects by analyzing the IHC expression score of these two proteins in the CCA tissue microarray. Correlative analysis revealed a positive correlation between CDK7 protein and MCL1 protein levels (Spearman’s rank = 0.39, *P* = 0.0002, Fig. 5c).

### Combined treatment of THZ1 with ABT-263 shows synergistic antitumor effects in CCA

Recent studies have demonstrated that MCL1, BCL2 and BCL-XL are key but independent determinants of cell survival^25^. To test this statement, we combined THZ1 treatment with a BCL2/BCL-XL inhibitor ABT-263 in HuCCT1 and HuH28 cell lines. Following 48 h of treatment, cell viability was assessed by CCK-8 assay and the CI was calculated based on the median-effect model of Chou–Talalay to determine whether the drug combination resulted in synergistic toxicity. As is evident from the heatmap representation of the CCK-8 assay, the combined treatment caused a strong inhibition of cell viability. This combination was synergistic in both HuCCT1 and HuH28 cell lines as CI was <1 (Fig. 5d). The synergistic apoptosis induction by the co-treatment of THZ1 and ABT-263 was also detected in HuCCT1 and HuH28 cell lines by the Annexin-V/PI assay, Caspase 3/7 activity assay, and cleaved PARP expression (Fig. 5e, f and Supplementary Fig. 5). Together, these results support that THZ1 synergizes with ABT-263 in inducing cell death in CCA.

### Genetic or pharmacological inhibition of MCL1 sensitizes CCA cells to ABT-263

**Fig. 6:**
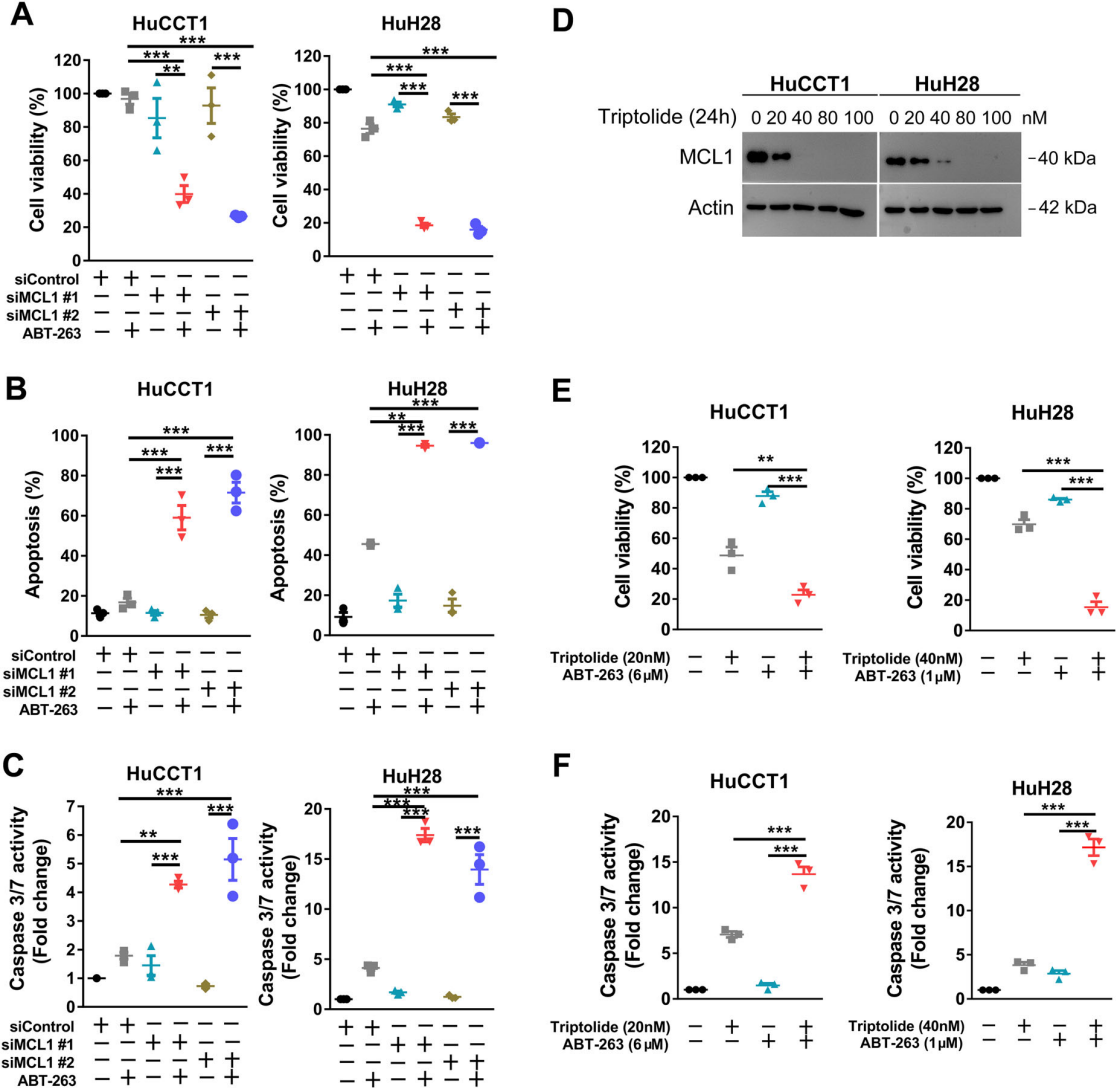
Repression of MCL1 shows similar effect to THZ1 in combination with ABT-263. **a** HuCCT1 and HuH28 cells were transfected with indicated siRNA for 48 h and then cultured in medium with or without ABT-263 at a concentration of 2 μM (HuCCT1) or 1 μM (HuH28) for another 48 h. Cell viability was measured by CCK-8 assay. Data represent mean ± SEM of three independent replicates (**P < 0.01; ***P < 0.001), **b**, **c** Cells were transfected with indicated siRNA for 48 h and then cultured in medium with or without ABT-263 at the above-mentioned concentrations for another 48 h. The apoptosis was detected by the Annexin-V/PI assay and Caspase 3/7 activity (**P < 0.01; ***P < 0.001). **d** Cells were treated with different concentrations of Triptolide for 24 h. MCL1 protein expression was analyzed by western blotting. **e**, **f** HuCCT1 and HuH28 cells were treated with Triptolide and ABT-263 at indicated concentrations for 48 h. Cell viability was measured by CCK-8 assay. Cell apoptosis was measured by Caspase 3/7 activity assay. Data represent mean ± SEM of three independent replicates. (**P < 0.01; ***P < 0.001)

Since our findings suggested that CDK7 inhibition may synergize with ABT-263 through downregulation of MCL1 expression, we next examined the function of MCL1 in the synergistic effect between THZ1 and ABT-263 in CCA cells. SiRNA mediated silencing of MCL1 resulted in great reduction in MCL1 protein levels and (Supplementary Fig. 6) potently enhanced the cell viability inhibition (Fig. 6a) and apoptosis induction (Fig. 6b, c and Supplementary Fig. 7) by ABT-263 in both HuCCT1 and HuH28 cell lines. Previous study has shown that the natural product Triptolide, the principle bioactive ingredient of the Chinese herb Tripterygium wilfordii Hook F, inhibited growth and downregulated MCL1 expression in CCA cells^29^. The results of western blotting validated the suppression of MCL1 by Triptolide in HuCCT1 and HuH28 cells (Fig. 6d). We extended our investigations to Triptolide and found that Triptolide had a similar combination effect with ABT-263 (Fig. 6e, f). Not surprisingly, A-1210477, a MCL1 selective inhibitor, also potentiated the antitumor effect of ABT-263 in CCA cells (Supplementary Fig. 8). Taken together, these data indicate that MCL1 played a key mechanism of action observed for the synergism between THZ1 and ABT-263.

### Knockdown of CDK7 or treatment with a new CDK7 inhibitor ICEC0942 leads to reduced cell viability and increases response to ABT-263

**Fig. 7:**
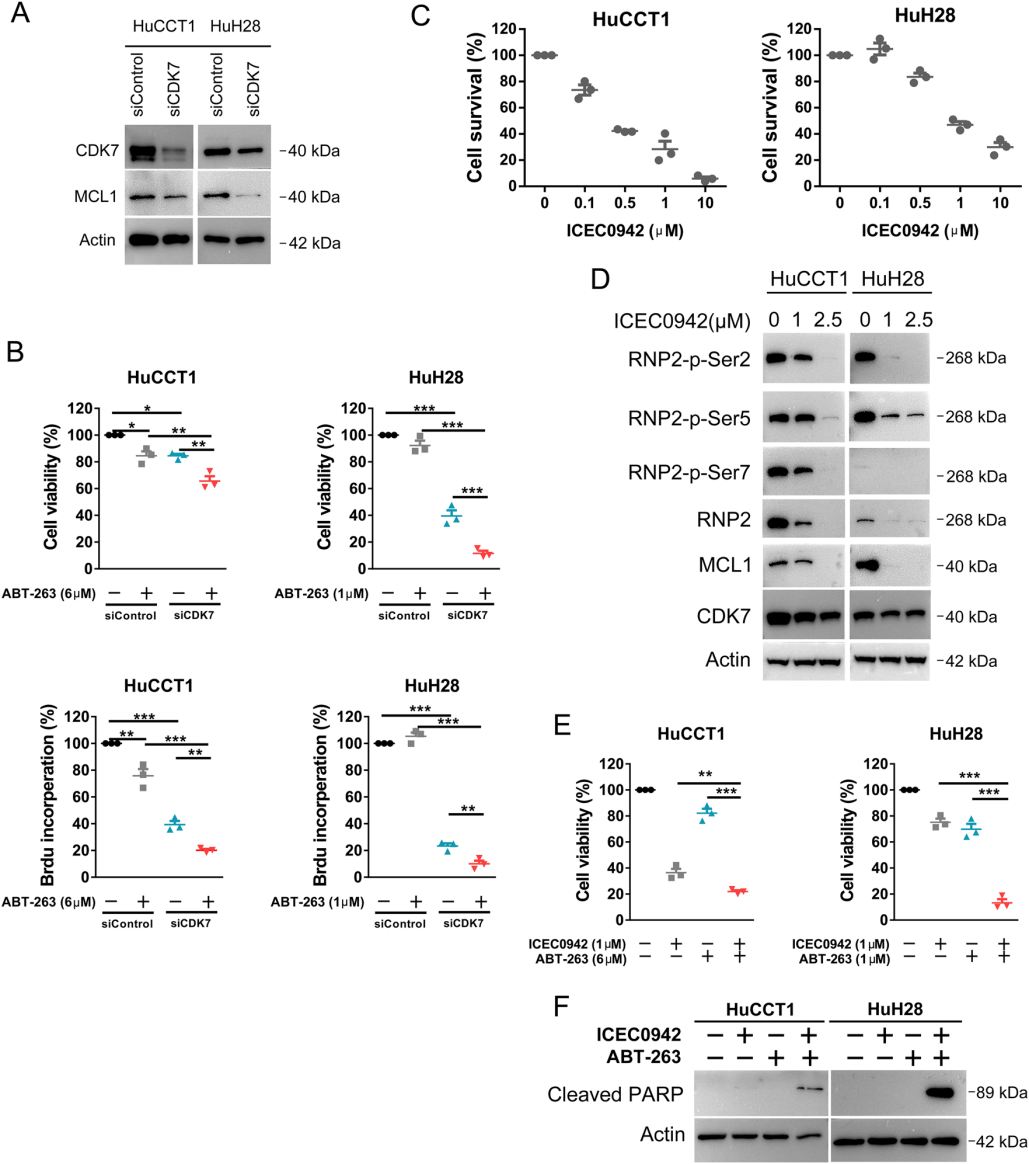
Repression of CDK7 inhibits CCA cells and sensitizes CCA cells to ABT-263. **a** HuCCT1 and HuH28 cells were transfected with indicated siRNA for 48 h. CDK7 and MCL1 protein expression was analyzed by western blotting. **b** HuCCT1 and HuH28 cells were exposed to ABT-263 at indicated concentration for 48 h after being transfected with indicated siRNA for 24 h. Cell viability was measured by CCK-8 assay and cell proliferation was detected using the BrdU proliferation ELISA kit. Data represent mean ± SEM of three independent replicates (*P < 0.05; **P < 0.01; ***P < 0.001). **c** HuCCT1 and HuH28 cells were treated with a novel CDK7 inhibitor ICEC0942 at different concentrations for 72 h. Cell viability was measured by CCK-8 assay. **d** HuCCT1 and HuH28 cells were exposed to ICEC0942 at indicated concentrations for 24 h. RNP2, RNP2 phosphorylation and MCL1 expression were analyzed by western blotting. **e** HuCCT1 and HuH28 cells were treated with ICEC0942 and ABT-263 at indicated concentrations for 48 h. Cell viability was measured by CCK-8 assay. Data represent mean ± SEM of three independent replicates. (*P < 0.05; **P < 0.01; ***P < 0.001). **f** HuCCT1 and HuH28 cells were treated with ICEC0942 and ABT-263 at above mentioned concentrations for 48 h. Cleaved PARP protein expression was analyzed by western blotting

To validate the function of CDK7 in CCA, we used siRNA to silence the expression of CDK7. After 48 h of transfection, CDK7 siRNA reduced CDK7 protein expression (Fig. 7a) and impaired cell viability and proliferation in both HuCCT1 and HuH28 cells (Fig. 7b). In addition, CDK7 siRNA reduced MCL1 protein expression and sensitized the effect of ABT-263 in both cell lines (Fig. 7a, b). Furthermore, ICEC0942, a new orally selective CDK7 small molecular inhibitor^30^, dose-dependently reduced cell viability in the two CCA cell lines tested (Fig. 7c). Treatment with ICEC0942 also caused a dose-dependent reduction of phosphorylation of RNP2 at S2, S5, and S7 (Fig. 7d), suggestive of RNA transcription inhibition through CDK7 inactivation. Similar to THZ1, ICEC0942 caused a reduction in MCL1 expression and potentiated the antitumor effect of ABT-263 in HuCCT1 and HuH28 cell lines (Fig. 7e). Combined treatment of ICEC0942 with ABT-263 caused strong apoptosis as evidenced by increased expression of cleaved PARP (Fig. 7f).

## Discussion

Although targeted therapies have revolutionized cancer treatment, CCA is still lack of effective targeted therapy. In this study, we screened a cell cycle compound library to search for novel therapeutic strategies for CCA and found CCA cells were sensitivity to CDK7 inhibition. CDK7 has been reported to play a crucial role in tumorigenesis. THZ1, as a CDK7 covalent inhibitor, is effective in several types of cancers, including esophageal squamous cell carcinoma, hepatocellular carcinoma, and so on. Here, we reported that THZ1 demonstrated antitumor effects in CCA through functional experiments both in vitro and in vivo. We also validated that interfering the expression of CDK7 by siRNA impaired cell viability and proliferation in CCA cell lines. These results suggested that CDK7 inhibition could be a potential therapeutic strategy for CCA.

To find molecular mechanism of the antitumor effects of THZ1 in CCA, we carried out RNA-Seq and found there were more than 1000 genes downregulated in three CCA cell lines after THZ1 treatment, including some well-known oncogenes such as SP1, FOSL1, RUNX1, and MCL1. Escaping apoptosis is crucial for the development and sustained growth of tumors. The BCL2 family of proteins is well known to be critical determinants of tumor cell survival, with CCA in particular having a high dependency on the pro-survival MCL1^26^. In CCA, it has been reported that MCL1 is often amplified and overexpressed and plays a crucial role in resistance to various therapeutic agents^26,31–35^. Its intrinsic structure makes it challenging to design effective targeted small molecules and few effective MCL1 inhibitors have entered clinical trials. In this regard, the availability of THZ1 will provide new opportunity to improve the treatment of CCA.

Cancer cells avoid apoptosis through various strategies that include increased expression of pro-survival proteins such as BCL-2, BCL-XL, or MCL-1. Targeting these proteins with BH3 mimetics has emerged as a promising strategy in cancer therapy. The first “on-target” BH3 mimetic, ABT-737^36^, and its orally bioavailable analog, ABT-263^37^, demonstrate broad-spectrum activity and inhibit BCL2, BCL-XL, and BCL-W but not MCL1. However, ABT-263 has shown limited efficacy in tumors with high MCL1 expression including CCA. In addition, ABT-263 causes thrombocytopenia due to on-target inhibition of BCL-XL in platelets.

Previous studies have shown that ABT-263 may be efficacious in some solid tumors when combined with other agents through decreasing MCL1 expression by transcriptional or post-translational mechanisms^38–43^. Since THZ1 could significantly repress the transcriptional expression of MCL1 without influencing the expression of BCL2 or BCL-XL in CCA, we hypothesized that it could act as a MCL1 inhibitor and synergize with ABT-263. As expected, we showed that the combination treatment caused potent apoptosis at low doses of both drugs compared to single-agent setting in CCA. This discovery may provide an improved therapeutic window which has significant clinical implications.

Super-enhancers (SEs) are large clusters of transcriptional enhancers, which show higher sensitivity to perturbation than typical enhancers. Cancer cells acquire SEs at oncogenes and other important genes for tumor pathogenesis. Thus, preferential disruption of SEs may provide new insights into inhibiting oncogenic drivers of cancer cells^5,13,15,16^. Prior studies indicate that SE-associated oncogenic transcripts can be significantly inhibited by THZ1 in a number of cancers, including nasopharyngeal carcinoma^15^, esophageal squamous cell carcinoma^13^, and hepatocellular carcinoma^16^. Whether and how SEs play a role in CCA biology remains unknown. Further studies are needed to establish the SE landscape in CCA cells, and verify that whether SE-associated oncogenes show exceptional sensitivity to THZ1 treatment, which may help further unveiling the antitumor mechanism of THZ1 in CCA.

In conclusion, our study provides preclinical evidence of CDK7 inhibition as a therapeutic strategy to treat CCA as monotherapy. In additionally, our results indicate that CDK7 inhibitor THZ1 can rapidly and dramatically inhibit MCL1 synthesis and drive apoptosis in combination with the BCL2/BCL-XL inhibitor in CCA.

## Supplementary information

Supplementary figure legend.

Supplementary Figure 1.

Supplementary Figure 2.

Supplementary figure 3.

Supplementary Figure 4.

Supplementary Figure 5.

Supplementary Figure 6.

Supplementary Figure 7.

Supplementary figure 8.

Supplementary table 1.

Supplementary table 2.

Supplementary table 3.

Supplementary table 4.

Supplementary table 5.

Supplementary table 6.

Supplementary table 7.

Supplementary table 8.

Supplementary table 9.
