# Supplementary material for: CDK7 inhibitor THZ1 inhibits MCL1 synthesis and drives cholangiocarcinoma apoptosis in combination with BCL2/BCL-XL inhibitor ABT-263
Source: Cell Death Dis. 2019 Aug 9;10(8):602. doi: 10.1038/s41419-019-1831-7 (PMC6688996; doi:10.1038/s41419-019-1831-7)
Supplement: Supplementary file 1 — Supplementary figure legend. [file 41419_2019_1831_MOESM1_ESM.docx]

Supplementary Fig.1. Five CCA cell lines (HuCCT1, HuH28, RBE, HCCC9810 and OZ) were plated in 96-well plates and treated with THZ1 at different concentrations for 72 h. Cell proliferation was measured by BrdU cell proliferation ELISA kit and bar graphs show percent cell proliferation to that of DMSO-treated cells. Data represents mean ± SEM of three dependent replicates.

Supplementary Fig.2. HuCCT1, HuH28 and RBE cells were treated with THZ1 at two concentrations (100 nM and 200 nM) for 72 h and cell apoptosis was measured by Annexin V-FITC/PI staining followed by flow cytometry.

Supplementary Fig.3. Nude mice were injected subcutaneously with HuCCT1 cells and randomly separated into two groups when tumor size reached around 200 mm^3^. Two groups of mice were treated with THZ1 (10 mg/kg, bid, i.p.) or vehicle (bid, i.p.) for 27 days, separately. Pictures of nude mice and tumors were shown.

Supplementary Fig.4. Mice weight were measured twice a week. The growth curve was shown.

Supplementary Fig.5. HuCCT1 and HuH28 cells were treated with THZ1 and ABT-263 for 48 h. The apoptosis was detected by the Annexin-V/PI assay.

Supplementary Fig.6. HuH28 and HuCCT1 were plated in 6-well plates and treated with siRNA for 48 h. MCL1 protein expression was analyzed by western blotting.

Supplementary Fig.7. Cells were transfected with indicated siRNA for 48 h and then cultured in medium with or without ABT-263 for another 48 h. The apoptosis was detected by the Annexin-V/PI assay.

Supplementary Fig.8. HuCCT1 and HuH28 were treated with A-1210477 and ABT263 at indicated concentrations for 48 h. Cell viability was measured by CCK-8 assay. Data represent mean±SEM of three dependent replicates. (**, P < 0.01; ***, P < 0.001)
