# Supplementary figures and images for: CDK7 inhibitor THZ1 inhibits MCL1 synthesis and drives cholangiocarcinoma apoptosis in combination with BCL2/BCL-XL inhibitor ABT-263
Source: Cell Death Dis. 2019 Aug 9;10(8):602. doi: 10.1038/s41419-019-1831-7 (PMC6688996; doi:10.1038/s41419-019-1831-7)

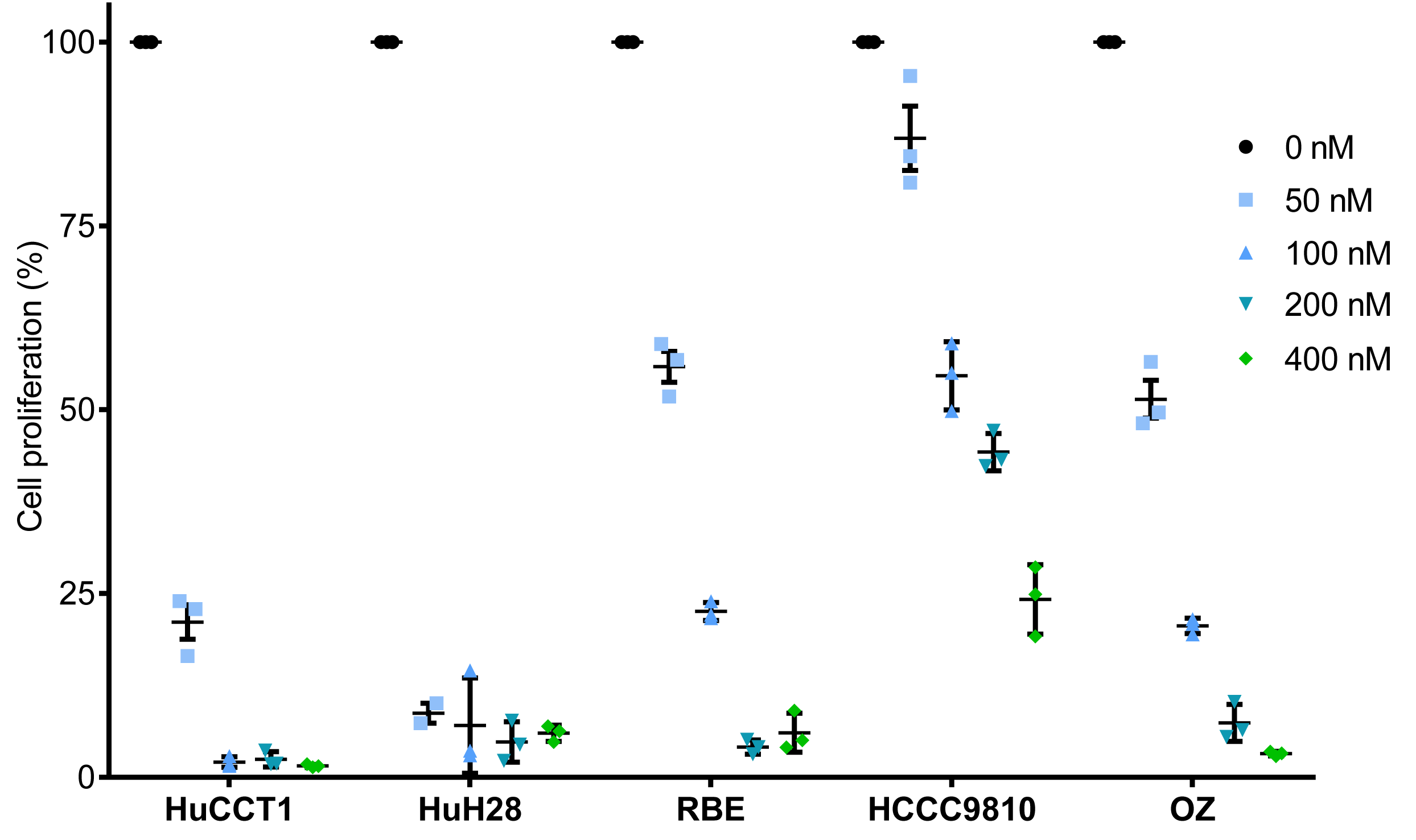

Supplement: Supplementary file 2 — Supplementary Figure 1. [file 41419_2019_1831_MOESM2_ESM.tif]

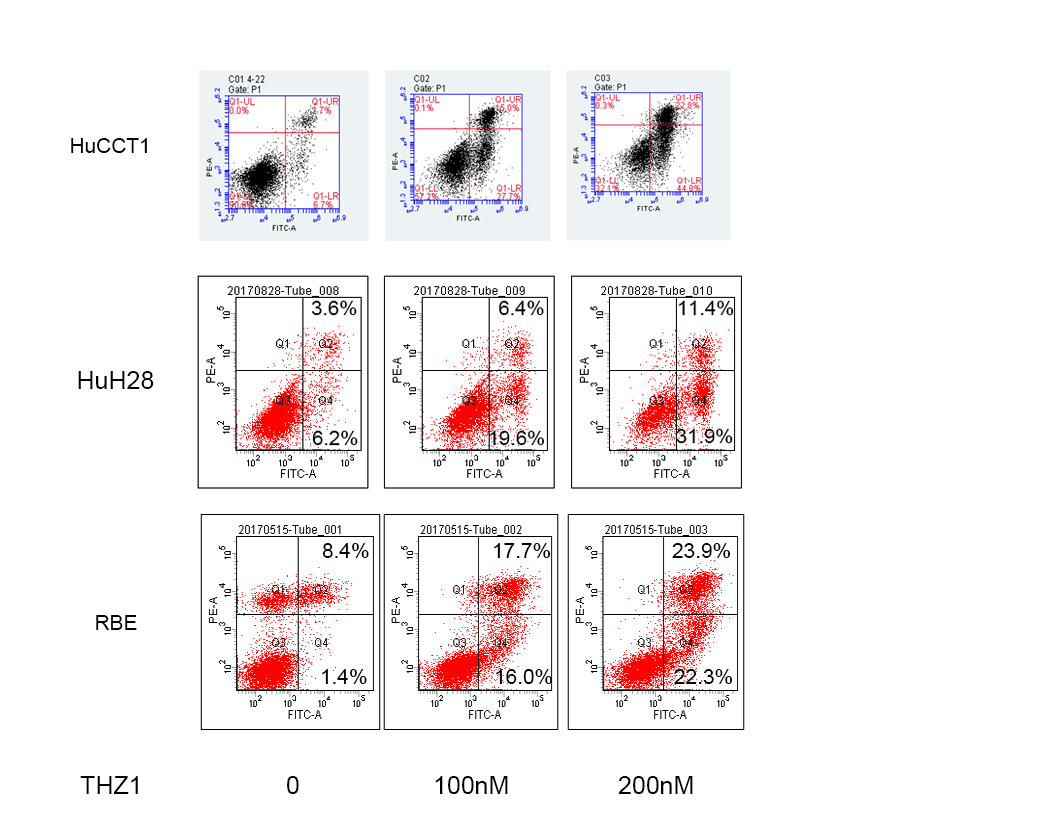

Supplement: Supplementary file 3 — Supplementary Figure 2. [file 41419_2019_1831_MOESM3_ESM.tif]

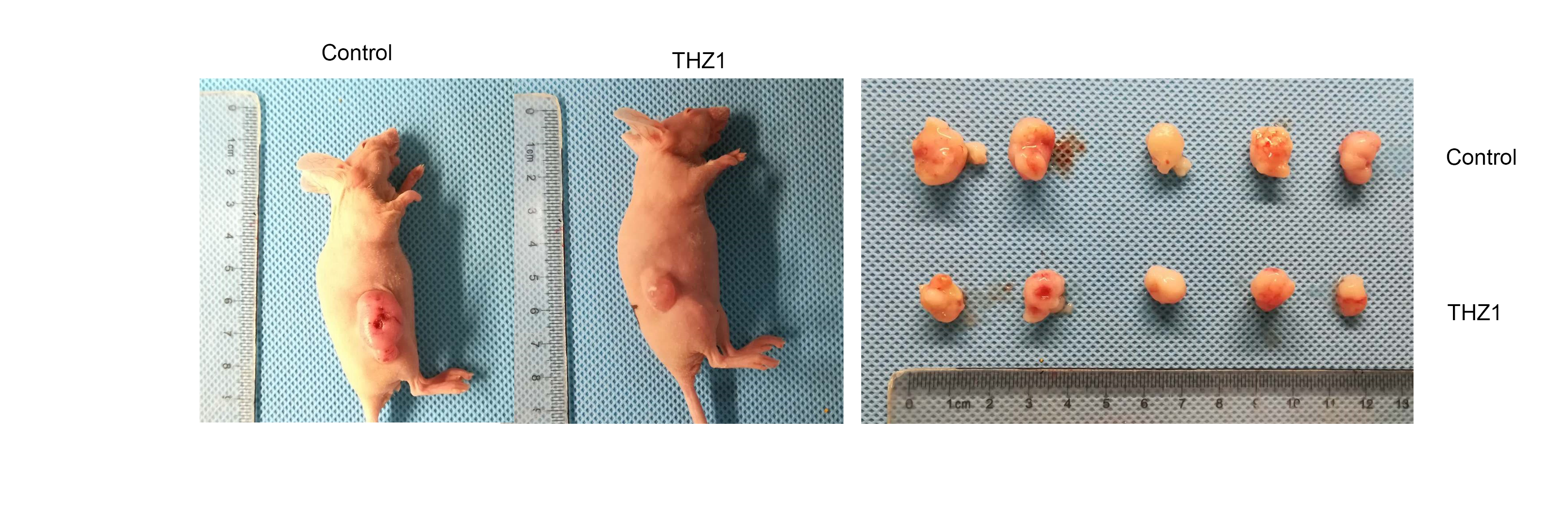

Supplement: Supplementary file 4 — Supplementary figure 3. [file 41419_2019_1831_MOESM4_ESM.tif]

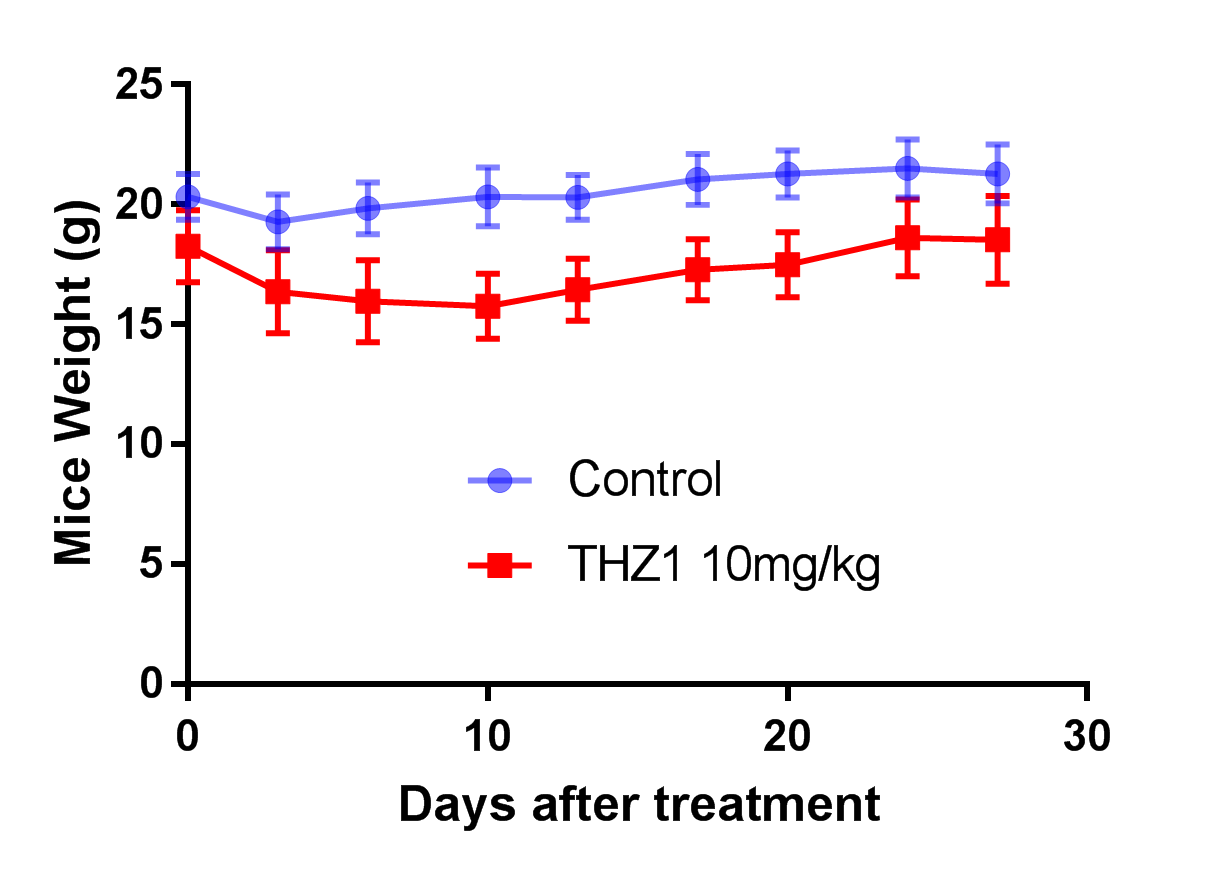

Supplement: Supplementary file 5 — Supplementary Figure 4. [file 41419_2019_1831_MOESM5_ESM.tif]

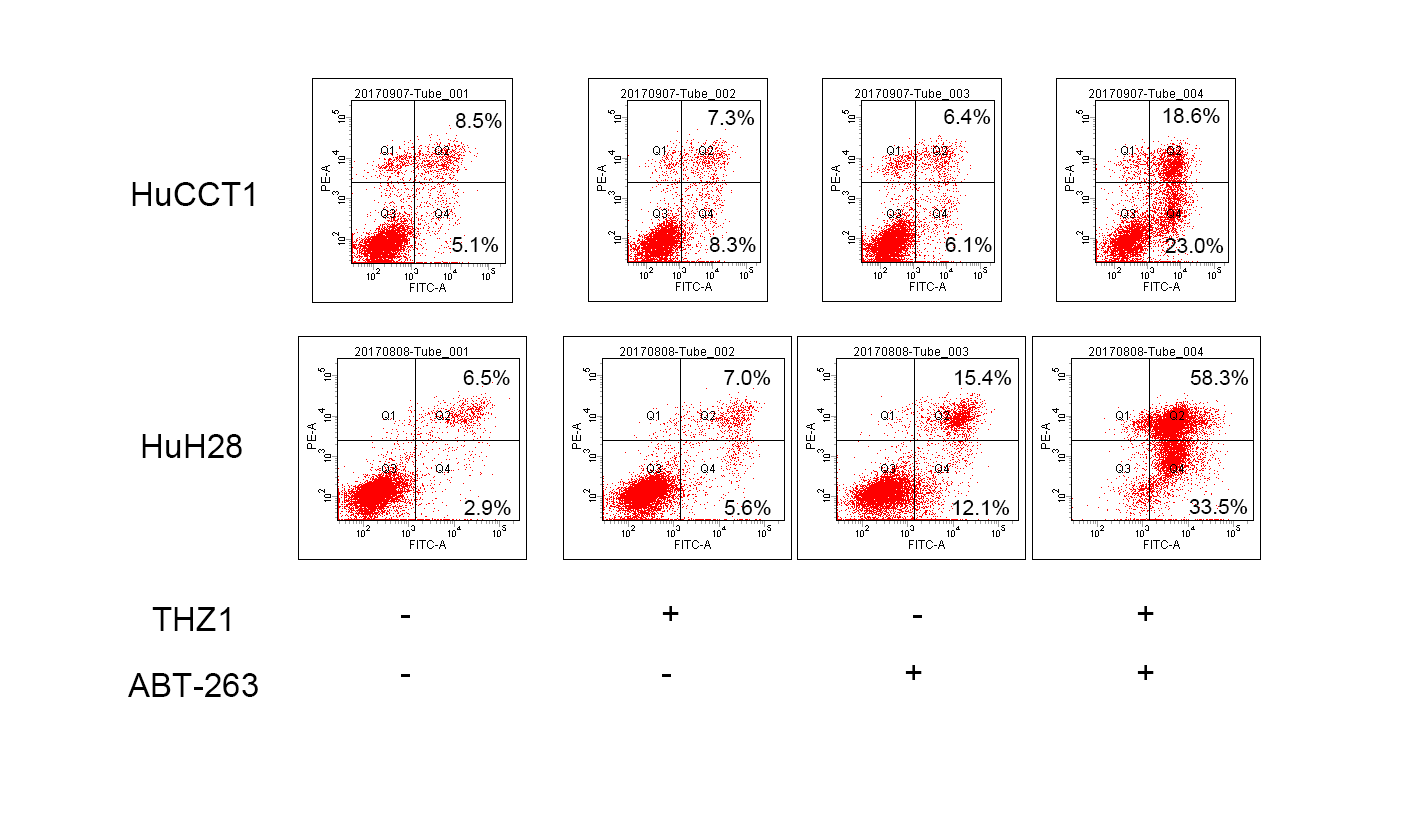

Supplement: Supplementary file 6 — Supplementary Figure 5. [file 41419_2019_1831_MOESM6_ESM.tif]

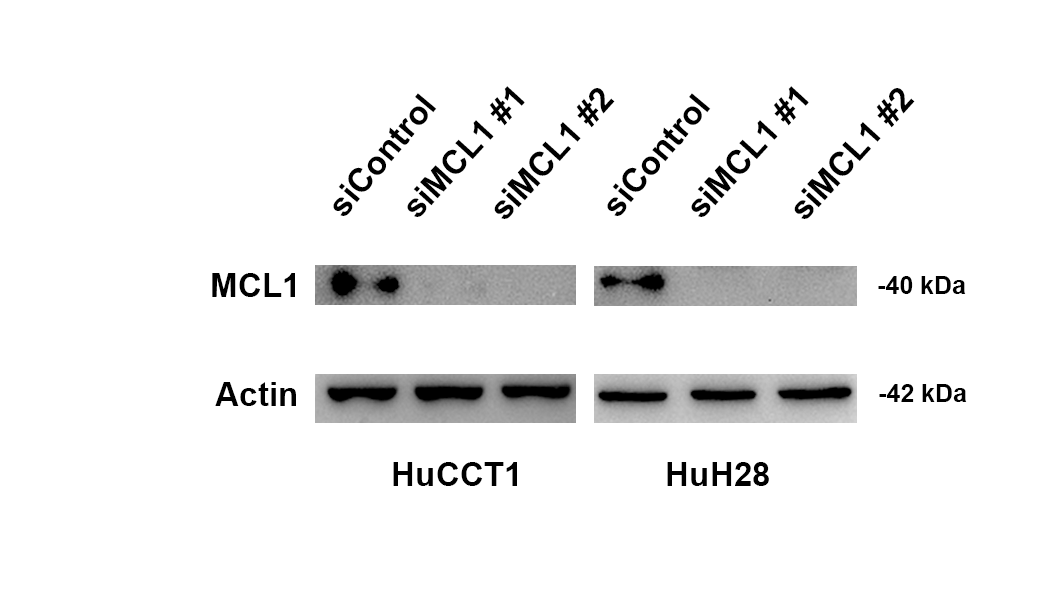

Supplement: Supplementary file 7 — Supplementary Figure 6. [file 41419_2019_1831_MOESM7_ESM.tif]

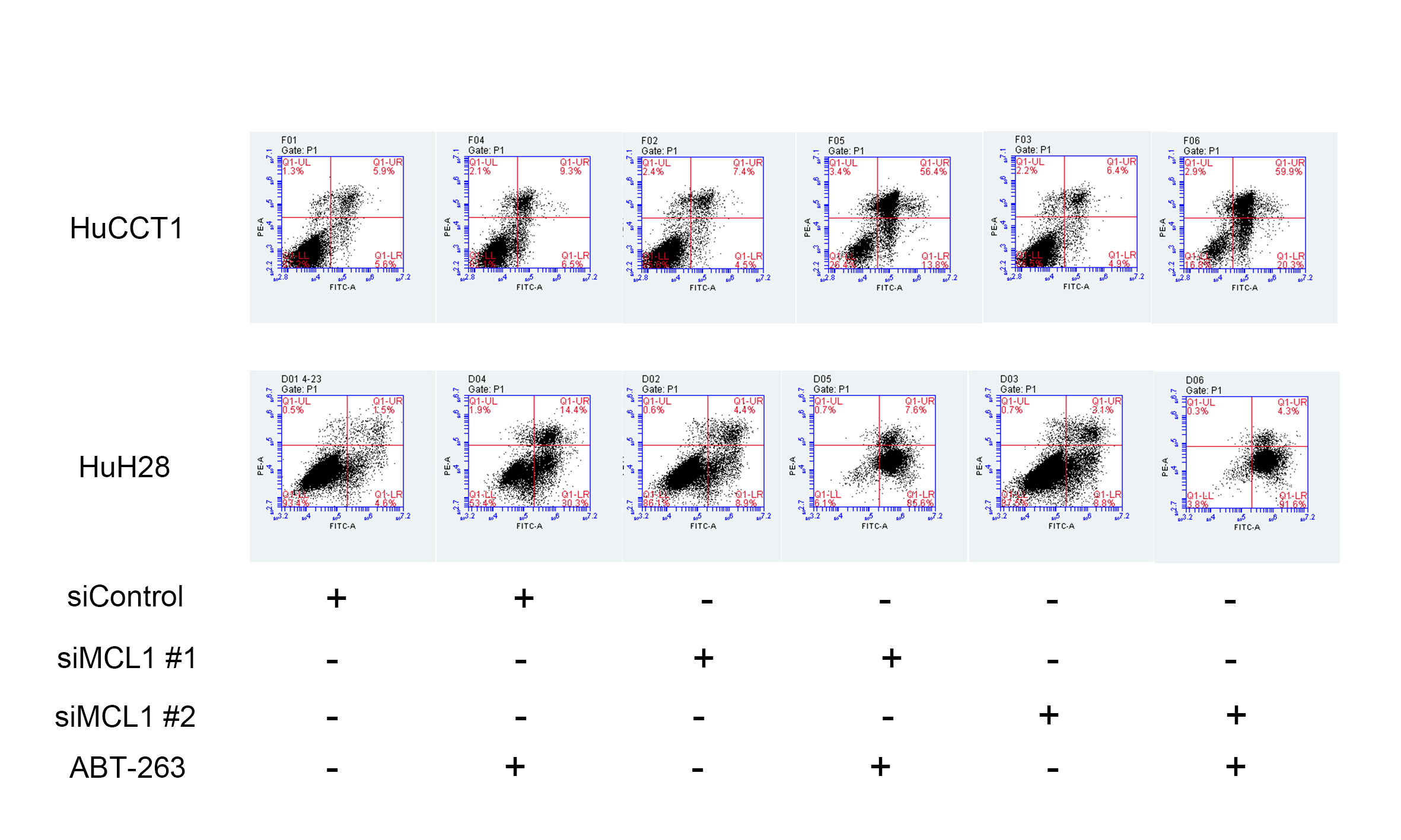

Supplement: Supplementary file 8 — Supplementary Figure 7. [file 41419_2019_1831_MOESM8_ESM.tif]

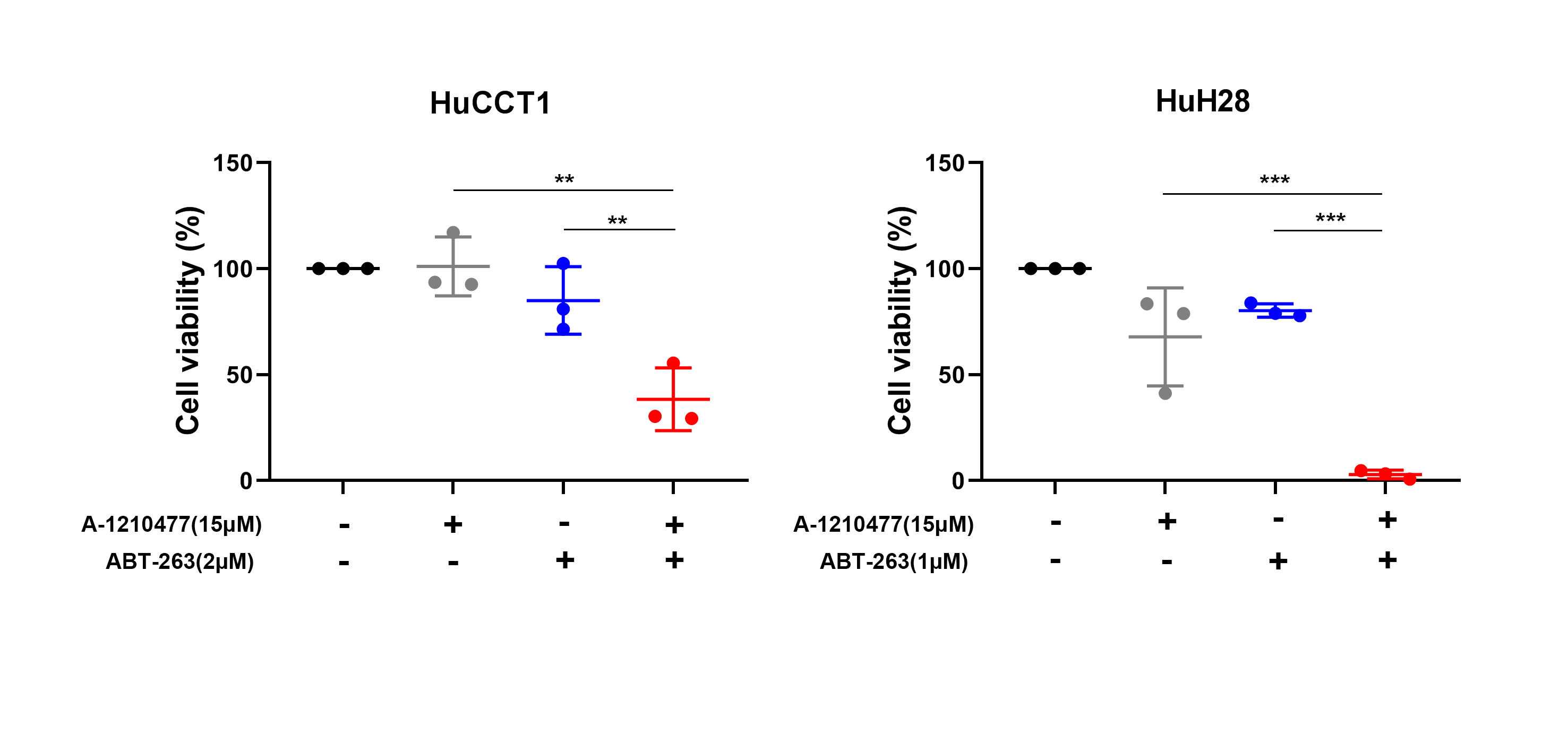

Supplement: Supplementary file 9 — Supplementary figure 8. [file 41419_2019_1831_MOESM9_ESM.tif]
