# Supplementary material for: CDK7 inhibitor THZ1 inhibits MCL1 synthesis and drives cholangiocarcinoma apoptosis in combination with BCL2/BCL-XL inhibitor ABT-263
Source: Cell Death Dis. 2019 Aug 9;10(8):602. doi: 10.1038/s41419-019-1831-7 (PMC6688996; doi:10.1038/s41419-019-1831-7)
Supplement: Supplementary file 10 — Supplementary table 1. [file 41419_2019_1831_MOESM10_ESM.docx]

Supplementary table 1. The sequences of primers for real-time qPCR

| Gene | Primer Sequence |
| --- | --- |
| MCL1 | Forward: CATTTCTTTTGGTGCCTTTGTG |
|  | Reverse: CCAGTCCCGTTTTGTCCTTAC |
| 18S | Forward: TTGGAGGGCAAGTCTGGTG |
|  | Reverse: CCGCTCCCAAGATCCAACTA |
