# Supplementary material for: CDK7 inhibitor THZ1 inhibits MCL1 synthesis and drives cholangiocarcinoma apoptosis in combination with BCL2/BCL-XL inhibitor ABT-263
Source: Cell Death Dis. 2019 Aug 9;10(8):602. doi: 10.1038/s41419-019-1831-7 (PMC6688996; doi:10.1038/s41419-019-1831-7)
Supplement: Supplementary file 11 — Supplementary table 2. [file 41419_2019_1831_MOESM11_ESM.docx]

| **Gene Name** | Target sequences |
| --- | --- |
| CDK7 | 5'-UUAAGGUUUCCACUGGACAGUUUGG-3' and |
|  | 5'-CCAAACUGUCCAGUGGAAACCUUAA-3' |
| MCL1 #1 | 5'-GAUUAUCUCUCGGUACCUUTT-3' and |
|  | 5'-AAGGUACCGAGAGAUAAUCTT-3' |
| MCL1 #2 | 5'-CGAAGGAAGUAUCGAAUUUTT-3' and |
|  | 5'-AAAUUCGAUACUUCCUUCGTT-3' |
| Negative Control | 5'-UUCUCCGAACGUGUCACGUTT-3' and |
|  | 5'-ACGUGACACGUUCGGAGAATT-3' |

Supplementary table 2. SiRNA Target sequences
