# Supplementary material for: CDK7 inhibitor THZ1 inhibits MCL1 synthesis and drives cholangiocarcinoma apoptosis in combination with BCL2/BCL-XL inhibitor ABT-263
Source: Cell Death Dis. 2019 Aug 9;10(8):602. doi: 10.1038/s41419-019-1831-7 (PMC6688996; doi:10.1038/s41419-019-1831-7)
Supplement: Supplementary file 16 — Supplementary table 7. [file 41419_2019_1831_MOESM16_ESM.pdf]

GO analysis of HUCCT1	
transcription, DNA-templated: 668 genes	regulation of transcription, DNA-templated:511 genes
ENSG00000131759	ENSG00000171316
ENSG00000171316	ENSG00000160685
ENSG00000167981	ENSG00000167981
ENSG00000183779	ENSG00000183779
ENSG00000099381	ENSG00000099381
ENSG00000151445	ENSG00000151445
ENSG00000170619	ENSG00000170619
ENSG00000109118	ENSG00000121417
ENSG00000121417	ENSG00000167840
ENSG00000167840	ENSG00000126778
ENSG00000251369	ENSG00000251369
ENSG00000176896	ENSG00000133884
ENSG00000133884	ENSG00000171425
ENSG00000171425	ENSG00000204524
ENSG00000197757	ENSG00000185219
ENSG00000204524	ENSG00000104903
ENSG00000185219	ENSG00000160679
ENSG00000104903	ENSG00000184939
ENSG00000184939	ENSG00000109787
ENSG00000169131	ENSG00000169131
ENSG00000109787	ENSG00000136870
ENSG00000136870	ENSG00000141646
ENSG00000141646	ENSG00000197857
ENSG00000197857	ENSG00000054598
ENSG00000113580	ENSG00000113580
ENSG00000141905	ENSG00000204366
ENSG00000204366	ENSG00000080298
ENSG00000080298	ENSG00000064195
ENSG00000156384	ENSG00000204920
ENSG00000204920	ENSG00000169957
ENSG00000109381	ENSG00000104221
ENSG00000196597	ENSG00000169955
ENSG00000139372	ENSG00000196597
ENSG00000117139	ENSG00000121406
ENSG00000197019	ENSG00000196152
ENSG00000170608	ENSG00000110851
ENSG00000198517	ENSG00000054267
ENSG00000196152	ENSG00000170604
ENSG00000121406	ENSG00000106261
ENSG00000132740	ENSG00000196150
ENSG00000054267	ENSG00000163795
ENSG00000170604	ENSG00000169951
ENSG00000196150	ENSG00000197937
ENSG00000106261	ENSG00000196793
ENSG00000163795	ENSG00000198315
ENSG00000169951	ENSG00000197841
ENSG00000156983	ENSG00000061936
ENSG00000137834	ENSG00000146278
ENSG00000067082	ENSG00000157429
ENSG00000148200	ENSG00000177374
ENSG00000197937	ENSG00000170260
ENSG00000196793	ENSG00000011332
ENSG00000198315	ENSG00000197024
ENSG00000104129	ENSG00000197128
ENSG00000197841	ENSG00000166949
ENSG00000160570	ENSG00000076108
ENSG00000159788	ENSG00000173480
ENSG00000061936	ENSG00000267680
ENSG00000186350	ENSG00000119574

ENSG00000124422	ENSG00000197935
ENSG00000146278	ENSG00000197933
ENSG00000157429	ENSG00000170265
ENSG00000170260	ENSG00000106031
ENSG00000177374	ENSG00000186280
ENSG00000197024	ENSG00000139651
ENSG00000011332	ENSG00000006704
ENSG00000197128	ENSG00000136574
ENSG00000103343	ENSG00000147124
ENSG00000166949	ENSG00000020256
ENSG00000173480	ENSG00000198604
ENSG00000076108	ENSG00000171448
ENSG00000267680	ENSG00000197037
ENSG00000169016	ENSG00000235109
ENSG00000167967	ENSG00000225614
ENSG00000119574	ENSG00000205903
ENSG00000197935	ENSG00000164463
ENSG00000100888	ENSG00000186272
ENSG00000170265	ENSG00000171456
ENSG00000197933	ENSG00000170631
ENSG00000106031	ENSG00000133639
ENSG00000186280	ENSG00000124444
ENSG00000157557	ENSG00000066422
ENSG00000139651	ENSG00000147548
ENSG00000006704	ENSG00000213096
ENSG00000179627	ENSG00000166860
ENSG00000198205	ENSG00000186376
ENSG00000140265	ENSG00000196670
ENSG00000186174	ENSG00000196466
ENSG00000057657	ENSG00000102349
ENSG00000033800	ENSG00000141956
ENSG00000147124	ENSG00000197044
ENSG00000020256	ENSG00000121454
ENSG00000197037	ENSG00000147118
ENSG00000171448	ENSG00000185252
ENSG00000198604	ENSG00000179119
ENSG00000204946	ENSG00000177683
ENSG00000235109	ENSG00000005801
ENSG00000225614	ENSG00000163946
ENSG00000205903	ENSG00000197714
ENSG00000164463	ENSG00000171469
ENSG00000186272	ENSG00000171466
ENSG00000144802	ENSG00000171467
ENSG00000204256	ENSG00000125285
ENSG00000171456	ENSG00000110395
ENSG00000156030	ENSG00000081386
ENSG00000170631	ENSG00000185650
ENSG00000124444	ENSG00000196458
ENSG00000066422	ENSG00000196456
ENSG00000147548	ENSG00000131845
ENSG00000213096	ENSG00000130684
ENSG00000166860	ENSG00000198551
ENSG00000186376	ENSG00000196453
ENSG00000196670	ENSG00000180626
ENSG00000164379	ENSG00000153207
ENSG00000186260	ENSG00000189308
ENSG00000102349	ENSG00000163935
ENSG00000196466	ENSG00000183647
ENSG00000141956	ENSG00000121903
ENSG00000165891	ENSG00000249471
ENSG00000197044	ENSG00000136997

ENSG00000121454	ENSG00000186026
ENSG00000147118	ENSG00000197162
ENSG00000185252	ENSG00000156853
ENSG00000179119	ENSG00000169155
ENSG00000177683	ENSG00000139718
ENSG00000005801	ENSG00000197062
ENSG00000163946	ENSG00000172216
ENSG00000135999	ENSG00000102804
ENSG00000197714	ENSG00000186020
ENSG00000171469	ENSG00000130856
ENSG00000171466	ENSG00000101457
ENSG00000125798	ENSG00000136715
ENSG00000171467	ENSG00000215421
ENSG00000204569	ENSG00000121931
ENSG00000131848	ENSG00000181896
ENSG00000198815	ENSG00000179922
ENSG00000125285	ENSG00000105136
ENSG00000158711	ENSG00000181894
ENSG00000081386	ENSG00000141568
ENSG00000196199	ENSG00000137843
ENSG00000196458	ENSG00000197961
ENSG00000196456	ENSG00000147789
ENSG00000131845	ENSG00000186017
ENSG00000130684	ENSG00000198538
ENSG00000198551	ENSG00000186130
ENSG00000196453	ENSG00000001167
ENSG00000180626	ENSG00000162676
ENSG00000155592	ENSG00000136866
ENSG00000116604	ENSG00000101442
ENSG00000189308	ENSG00000267508
ENSG00000163935	ENSG00000007968
ENSG00000183647	ENSG00000105127
ENSG00000121903	ENSG00000055609
ENSG00000177485	ENSG00000101544
ENSG00000037965	ENSG00000254004
ENSG00000249471	ENSG00000155545
ENSG00000178764	ENSG00000101665
ENSG00000101945	ENSG00000181315
ENSG00000186026	ENSG00000183309
ENSG00000197162	ENSG00000135747
ENSG00000141026	ENSG00000172171
ENSG00000087152	ENSG00000180479
ENSG00000156853	ENSG00000152439
ENSG00000169155	ENSG00000152433
ENSG00000139718	ENSG00000196428
ENSG00000197063	ENSG00000135164
ENSG00000197062	ENSG00000135365
ENSG00000186141	ENSG00000152784
ENSG00000170365	ENSG00000197483
ENSG00000172216	ENSG00000116833
ENSG00000196588	ENSG00000116731
ENSG00000186020	ENSG00000170949
ENSG00000169925	ENSG00000196705
ENSG00000130856	ENSG00000165655
ENSG00000101457	ENSG00000048649
ENSG00000077684	ENSG00000185697
ENSG00000136715	ENSG00000152443
ENSG00000129911	ENSG00000172845
ENSG00000215421	ENSG00000105556
ENSG00000121931	ENSG00000171940
ENSG00000181896	ENSG00000196417

ENSG00000105136	ENSG00000165512
ENSG00000179922	ENSG00000170100
ENSG00000170684	ENSG00000198879
ENSG00000181894	ENSG00000174197
ENSG00000102984	ENSG00000143842
ENSG00000197961	ENSG00000205189
ENSG00000112200	ENSG00000181638
ENSG00000147789	ENSG00000130803
ENSG00000198538	ENSG00000155760
ENSG00000186017	ENSG00000019485
ENSG00000186130	ENSG00000171970
ENSG00000048052	ENSG00000172977
ENSG00000141570	ENSG00000177463
ENSG00000185129	ENSG00000169184
ENSG00000162676	ENSG00000116990
ENSG00000136866	ENSG00000130818
ENSG00000101442	ENSG00000016082
ENSG00000267508	ENSG00000025293
ENSG00000150907	ENSG00000178691
ENSG00000118482	ENSG00000061273
ENSG00000196843	ENSG00000218891
ENSG00000055609	ENSG00000213799
ENSG00000105127	ENSG00000185670
ENSG00000101544	ENSG00000172262
ENSG00000166135	ENSG00000204859
ENSG00000254004	ENSG00000151014
ENSG00000155545	ENSG00000118260
ENSG00000101665	ENSG00000091656
ENSG00000181315	ENSG00000213588
ENSG00000183309	ENSG00000129474
ENSG00000143867	ENSG00000171574
ENSG00000180479	ENSG00000171988
ENSG00000152439	ENSG00000155508
ENSG00000152433	ENSG00000010539
ENSG00000135164	ENSG00000189079
ENSG00000135365	ENSG00000164048
ENSG00000152784	ENSG00000148300
ENSG00000013619	ENSG00000172273
ENSG00000197483	ENSG00000169981
ENSG00000116833	ENSG00000173276
ENSG00000095787	ENSG00000078246
ENSG00000163159	ENSG00000173275
ENSG00000116731	ENSG00000125482
ENSG00000170949	ENSG00000171161
ENSG00000196705	ENSG00000171163
ENSG00000165655	ENSG00000011258
ENSG00000148143	ENSG00000140382
ENSG00000152443	ENSG00000197782
ENSG00000234602	ENSG00000165417
ENSG00000172845	ENSG00000196757
ENSG00000105556	ENSG00000182986
ENSG00000182742	ENSG00000166188
ENSG00000179456	ENSG00000196652
ENSG00000171940	ENSG00000105497
ENSG00000196417	ENSG00000267041
ENSG00000135457	ENSG00000122386
ENSG00000165512	ENSG00000182318
ENSG00000168395	ENSG00000169375
ENSG00000170100	ENSG00000177200
ENSG00000145041	ENSG00000173120
ENSG00000068654	ENSG00000185591

ENSG00000105229	ENSG00000122482
ENSG00000173011	ENSG00000197385
ENSG00000174197	ENSG00000198728
ENSG00000143842	ENSG00000204604
ENSG00000205189	ENSG00000097007
ENSG00000078403	ENSG00000196781
ENSG00000181638	ENSG00000112584
ENSG00000130803	ENSG00000124201
ENSG00000079432	ENSG00000213762
ENSG00000019485	ENSG00000181220
ENSG00000171970	ENSG00000197279
ENSG00000172977	ENSG00000189266
ENSG00000177463	ENSG00000204519
ENSG00000163635	ENSG00000100395
ENSG00000117713	ENSG00000006432
ENSG00000078900	ENSG00000214029
ENSG00000169184	ENSG00000117000
ENSG00000108175	ENSG00000106479
ENSG00000096717	ENSG00000177426
ENSG00000169635	ENSG00000164011
ENSG00000166261	ENSG00000204611
ENSG00000130818	ENSG00000103199
ENSG00000025293	ENSG00000158636
ENSG00000178691	ENSG00000181555
ENSG00000148737	ENSG00000152518
ENSG00000061273	ENSG00000204514
ENSG00000218891	ENSG00000102870
ENSG00000213799	ENSG00000160094
ENSG00000185670	ENSG00000175213
ENSG00000172262	ENSG00000156650
ENSG00000204859	ENSG00000117751
ENSG00000198890	ENSG00000163877
ENSG00000091656	ENSG00000197363
ENSG00000198894	ENSG00000163872
ENSG00000117036	ENSG00000197362
ENSG00000189298	ENSG00000148835
ENSG00000118263	ENSG00000171295
ENSG00000213588	ENSG00000134138
ENSG00000129474	ENSG00000146587
ENSG00000171574	ENSG00000028839
ENSG00000059728	ENSG00000173041
ENSG00000171988	ENSG00000160199
ENSG00000132275	ENSG00000071243
ENSG00000155508	ENSG00000161914
ENSG00000010539	ENSG00000168310
ENSG00000030419	ENSG00000263002
ENSG00000189079	ENSG00000149050
ENSG00000172273	ENSG00000115289
ENSG00000162714	ENSG00000160062
ENSG00000197256	ENSG00000168813
ENSG00000169981	ENSG00000178229
ENSG00000173276	ENSG00000009954
ENSG00000173275	ENSG00000153767
ENSG00000171161	ENSG00000113658
ENSG00000171163	ENSG00000005339
ENSG00000125484	ENSG00000198093
ENSG00000128604	ENSG00000167562
ENSG00000011258	ENSG00000137338
ENSG00000172818	ENSG00000086712
ENSG00000179588	ENSG00000167565
ENSG00000116044	ENSG00000168286

ENSG00000140382	ENSG00000171634
ENSG00000197782	ENSG00000159882
ENSG00000174282	ENSG00000143157
ENSG00000196757	ENSG00000159885
ENSG00000182986	ENSG00000148400
ENSG00000261221	ENSG00000048405
ENSG00000166188	ENSG00000084093
ENSG00000196652	ENSG00000189042
ENSG00000105497	ENSG00000173068
ENSG00000162711	ENSG00000133250
ENSG00000267041	ENSG00000120832
ENSG00000122386	ENSG00000010818
ENSG00000182318	ENSG00000036549
ENSG00000169375	ENSG00000124813
ENSG00000180530	ENSG00000160993
ENSG00000116580	ENSG00000070495
ENSG00000177200	ENSG00000133247
ENSG00000173120	ENSG00000134107
ENSG00000132005	ENSG00000163349
ENSG00000122482	ENSG00000146757
ENSG00000197385	ENSG00000161526
ENSG00000198728	ENSG00000075292
ENSG00000204604	ENSG00000178386
ENSG00000162601	ENSG00000168556
ENSG00000172943	ENSG00000158691
ENSG00000196781	ENSG00000124151
ENSG00000112584	ENSG00000187626
ENSG00000163166	ENSG00000197566
ENSG00000148297	ENSG00000189120
ENSG00000213762	ENSG00000101096
ENSG00000148840	ENSG00000142528
ENSG00000183337	ENSG00000188785
ENSG00000181220	ENSG00000205683
ENSG00000013503	ENSG00000234444
ENSG00000197279	ENSG00000185278
ENSG00000155666	ENSG00000118707
ENSG00000224470	ENSG00000198182
ENSG00000204519	ENSG00000131931
ENSG00000189266	ENSG00000096654
ENSG00000100395	ENSG00000112365
ENSG00000006432	ENSG00000137574
ENSG00000117000	ENSG00000161551
ENSG00000214029	ENSG00000168564
ENSG00000106479	ENSG00000168661
ENSG00000177426	ENSG00000124160
ENSG00000204611	ENSG00000184677
ENSG00000221869	ENSG00000142409
ENSG00000148411	ENSG00000269343
ENSG00000103199	ENSG00000172493
ENSG00000181690	ENSG00000142556
ENSG00000135111	ENSG00000152382
ENSG00000171681	ENSG00000131115
ENSG00000158636	ENSG00000118412
ENSG00000137504	ENSG00000198298
ENSG00000160094	ENSG00000173875
ENSG00000102870	ENSG00000143067
ENSG00000156650	ENSG00000143970
ENSG00000175213	ENSG00000155846
ENSG00000204713	ENSG00000006194
ENSG00000117751	ENSG00000176024
ENSG00000197363	ENSG00000187607

ENSG00000197362	ENSG00000171606
ENSG00000171295	ENSG00000152475
ENSG00000143614	ENSG00000143373
ENSG00000197323	ENSG00000187792
ENSG00000146587	ENSG00000115568
ENSG00000171056	ENSG00000114853
ENSG00000173041	ENSG00000196700
ENSG00000071243	ENSG00000107104
ENSG00000161914	ENSG00000064932
ENSG00000168826	ENSG00000144747
ENSG00000113645	ENSG00000185024
ENSG00000078699	ENSG00000198026
ENSG00000263002	ENSG00000100207
ENSG00000149050	ENSG00000158161
ENSG00000173894	ENSG00000159917
ENSG00000115289	ENSG00000175691
ENSG00000160062	ENSG00000143498
ENSG00000168813	ENSG00000125945
ENSG00000178229	ENSG00000124782
ENSG00000043355	ENSG00000175197
ENSG00000009954	ENSG00000166526
ENSG00000070476	ENSG00000166529
ENSG00000113658	ENSG00000125846
ENSG00000198093	ENSG00000125845
ENSG00000148606	ENSG00000213020
ENSG00000019549	ENSG00000101040
ENSG00000167562	ENSG00000083817
ENSG00000086712	ENSG00000083812
ENSG00000167565	ENSG00000196812
ENSG00000184436	ENSG00000157657
ENSG00000168286	ENSG00000138767
ENSG00000171634	ENSG00000140836
ENSG00000159882	ENSG00000168795
ENSG00000159885	ENSG00000180855
ENSG00000048405	ENSG00000204209
ENSG00000084093	ENSG00000111596
ENSG00000189042	ENSG00000137075
ENSG00000173068	ENSG00000196345
ENSG00000159388	ENSG00000136504
ENSG00000133250	ENSG00000151612
ENSG00000056277	ENSG00000186918
ENSG00000120832	ENSG00000083828
ENSG00000036549	ENSG00000107882
ENSG00000160993	ENSG00000020577
ENSG00000070495	ENSG00000196110
ENSG00000068024	ENSG00000167384
ENSG00000133247	ENSG00000198455
ENSG00000134107	ENSG00000185947
ENSG00000163349	ENSG00000177045
ENSG00000008083	ENSG00000137185
ENSG00000146757	ENSG00000130544
ENSG00000161526	ENSG00000256087
ENSG00000162702	ENSG00000131051
ENSG00000176396	ENSG00000196214
ENSG00000075292	ENSG00000198040
ENSG00000178386	ENSG00000151090
ENSG00000168062	ENSG00000178935
ENSG00000140577	ENSG00000167657
ENSG00000168556	ENSG00000144354
ENSG00000058600	ENSG00000256294
ENSG00000135334	ENSG00000203326

ENSG00000071282	ENSG00000180884
ENSG00000158691	ENSG00000127663
ENSG00000124151	ENSG00000160908
ENSG00000184402	ENSG00000179943
ENSG00000187626	ENSG00000067646
ENSG00000197566	ENSG00000167395
ENSG00000101191	ENSG00000170325
ENSG00000189120	ENSG00000198466
ENSG00000123411	ENSG00000167394
ENSG00000063978	ENSG00000170322
ENSG00000188785	ENSG00000186812
ENSG00000205683	ENSG00000167785
ENSG00000234444	ENSG00000079999
ENSG00000118707	ENSG00000183621
ENSG00000185278	ENSG00000100105
ENSG00000198182	ENSG00000088876
ENSG00000131931	ENSG00000100109
ENSG00000096654	ENSG00000081189
ENSG00000115641	ENSG00000114933
ENSG00000137574	ENSG00000177873
ENSG00000112365	ENSG00000126746
ENSG00000065526	ENSG00000105732
ENSG00000161551	ENSG00000174652
ENSG00000125812	ENSG00000197619
ENSG00000124160	ENSG00000167637
ENSG00000168661	ENSG00000177125
ENSG00000143190	ENSG00000196378
ENSG00000184677	ENSG00000167635
ENSG00000142409	ENSG00000185730
ENSG00000143258	ENSG00000063438
ENSG00000115548	ENSG00000131263
ENSG00000129315	ENSG00000176476
ENSG00000142556	ENSG00000198105
ENSG00000198081	ENSG00000183742
ENSG00000101076	ENSG00000005889
ENSG00000152382	ENSG00000131061
ENSG00000131115	ENSG00000113812
ENSG00000118412	ENSG00000178917
ENSG00000140548	ENSG00000114315
ENSG00000114861	ENSG00000124459
ENSG00000198298	ENSG00000151623
ENSG00000173875	ENSG00000180035
ENSG00000174306	ENSG00000186448
ENSG00000143067	ENSG00000186300
ENSG00000143970	ENSG00000140987
ENSG00000006194	ENSG00000167034
ENSG00000205659	ENSG00000167625
ENSG00000157933	ENSG00000196387
ENSG00000143379	ENSG00000112983
ENSG00000176024	ENSG00000083168
ENSG00000164916	ENSG00000123095
ENSG00000187607	ENSG00000105997
ENSG00000171606	ENSG00000204335
ENSG00000152475	ENSG00000118620
ENSG00000143373	ENSG00000144597
ENSG00000187792	ENSG00000140332
ENSG00000171604	ENSG00000111424
ENSG00000167685	ENSG00000105619
ENSG00000165244	ENSG00000196357
ENSG00000115568	ENSG00000198393
ENSG00000114853	ENSG00000105991

ENSG00000196700	ENSG00000140992
ENSG00000107104	ENSG00000123636
ENSG00000080603	ENSG00000124766
ENSG00000064932	ENSG00000085185
ENSG00000109971	ENSG00000196391
ENSG00000128908	ENSG00000089335
ENSG00000179833	ENSG00000164442
ENSG00000198026	ENSG00000144791
ENSG00000100207	ENSG00000236104
ENSG00000100201	ENSG00000117222
ENSG00000158161	ENSG00000177842
ENSG00000159917	ENSG00000077092
ENSG00000175691	ENSG00000167548
ENSG00000125945	ENSG00000164631
ENSG00000256060	ENSG00000127989
ENSG00000168517	ENSG00000136630
ENSG00000178951	ENSG00000197608
ENSG00000175197	ENSG00000180938
ENSG00000170689	ENSG00000085276
ENSG00000172466	ENSG00000062370
ENSG00000117318	ENSG00000085274
ENSG00000167771	ENSG00000100625
ENSG00000166526	ENSG00000101126
ENSG00000166529	ENSG00000105708
ENSG00000150347	ENSG00000187815
ENSG00000125846	
ENSG00000204304	
ENSG00000213020	
ENSG00000198146	
ENSG00000083817	
ENSG00000083812	
ENSG00000175745	
ENSG00000105821	
ENSG00000120616	
ENSG00000113916	
ENSG00000157657	
ENSG00000196812	
ENSG00000124496	
ENSG00000140320	
ENSG00000138767	
ENSG00000168795	
ENSG00000165671	
ENSG00000180855	
ENSG00000204209	
ENSG00000213024	
ENSG00000111596	
ENSG00000196345	
ENSG00000136504	
ENSG00000186416	
ENSG00000151612	
ENSG00000066135	
ENSG00000083828	
ENSG00000186918	
ENSG00000215271	
ENSG00000089902	
ENSG00000119608	
ENSG00000196110	
ENSG00000167384	
ENSG00000185947	
ENSG00000198455	
ENSG00000177045	

ENSG00000137185	
ENSG00000130544	
ENSG00000256087	
ENSG00000125968	
ENSG00000131051	
ENSG00000196214	
ENSG00000198040	
ENSG00000151090	
ENSG00000178935	
ENSG00000167657	
ENSG00000149308	
ENSG00000144354	
ENSG00000112242	
ENSG00000256294	
ENSG00000104064	
ENSG00000203326	
ENSG00000180884	
ENSG00000083838	
ENSG00000160908	
ENSG00000127663	
ENSG00000141582	
ENSG00000067646	
ENSG00000179943	
ENSG00000167395	
ENSG00000170325	
ENSG00000167394	
ENSG00000198466	
ENSG00000186812	
ENSG00000167785	
ENSG00000079999	
ENSG00000183814	
ENSG00000183621	
ENSG00000088876	
ENSG00000100105	
ENSG00000081189	
ENSG00000114933	
ENSG00000177873	
ENSG00000005483	
ENSG00000126368	
ENSG00000105732	
ENSG00000126746	
ENSG00000139154	
ENSG00000174652	
ENSG00000197619	
ENSG00000167637	
ENSG00000177125	
ENSG00000196378	
ENSG00000160633	
ENSG00000185730	
ENSG00000063438	
ENSG00000183741	
ENSG00000131263	
ENSG00000105866	
ENSG00000176476	
ENSG00000198105	
ENSG00000167528	
ENSG00000183742	
ENSG00000005889	
ENSG00000179862	
ENSG00000104881	
ENSG00000131061	

ENSG00000153922	
ENSG00000113812	
ENSG00000197905	
ENSG00000114315	
ENSG00000151623	
ENSG00000180035	
ENSG00000186448	
ENSG00000186300	
ENSG00000140987	
ENSG00000167034	
ENSG00000176678	
ENSG00000167625	
ENSG00000105722	
ENSG00000196387	
ENSG00000112983	
ENSG00000121671	
ENSG00000186834	
ENSG00000176182	
ENSG00000166886	
ENSG00000083168	
ENSG00000049246	
ENSG00000177853	
ENSG00000123095	
ENSG00000105997	
ENSG00000204335	
ENSG00000118620	
ENSG00000144597	
ENSG00000140332	
ENSG00000111424	
ENSG00000105619	
ENSG00000198393	
ENSG00000196357	
ENSG00000105991	
ENSG00000140992	
ENSG00000123636	
ENSG00000105085	
ENSG00000085185	
ENSG00000196391	
ENSG00000089335	
ENSG00000164442	
ENSG00000178409	
ENSG00000144791	
ENSG00000236104	
ENSG00000117222	
ENSG00000177842	
ENSG00000077092	
ENSG00000167548	
ENSG00000164631	
ENSG00000136630	
ENSG00000127528	
ENSG00000197608	
ENSG00000180938	
ENSG00000085276	
ENSG00000062370	
ENSG00000085274	
ENSG00000101126	
ENSG00000172530	
ENSG00000101255	
ENSG00000105708	
ENSG00000187815	
