# Supplementary material for: CDK7 inhibitor THZ1 inhibits MCL1 synthesis and drives cholangiocarcinoma apoptosis in combination with BCL2/BCL-XL inhibitor ABT-263
Source: Cell Death Dis. 2019 Aug 9;10(8):602. doi: 10.1038/s41419-019-1831-7 (PMC6688996; doi:10.1038/s41419-019-1831-7)
Supplement: Supplementary file 17 — Supplementary table 8. [file 41419_2019_1831_MOESM17_ESM.pdf]

GO analysis of HuH28	
transcription, DNA-templated: 263 genes	egulation of transcription, DNA-templated: 219 genes
ENSG00000146587	ENSG00000146587
ENSG00000131759	ENSG00000173041
ENSG00000173041	ENSG00000167981
ENSG00000167981	ENSG00000161914
ENSG00000168826	ENSG00000099381
ENSG00000161914	ENSG00000167840
ENSG00000099381	ENSG00000251369
ENSG00000167840	ENSG00000171425
ENSG00000251369	ENSG00000185219
ENSG00000171425	ENSG00000168310
ENSG00000185219	ENSG00000263002
ENSG00000263002	ENSG00000160679
ENSG00000184939	ENSG00000184939
ENSG00000109787	ENSG00000109787
ENSG00000136870	ENSG00000136870
ENSG00000197857	ENSG00000197857
ENSG00000204366	ENSG00000204366
ENSG00000173894	ENSG00000204920
ENSG00000156384	ENSG00000104221
ENSG00000204920	ENSG00000169955
ENSG00000178229	ENSG00000178229
ENSG00000196152	ENSG00000153767
ENSG00000121406	ENSG00000196152
ENSG00000170604	ENSG00000121406
ENSG00000148606	ENSG00000110851
ENSG00000106261	ENSG00000170604
ENSG00000198093	ENSG00000106261
ENSG00000196150	ENSG00000198093
ENSG00000169951	ENSG00000196150
ENSG00000167562	ENSG00000169951
ENSG00000197937	ENSG00000167562
ENSG00000198315	ENSG00000197937
ENSG00000197841	ENSG00000198315
ENSG00000184436	ENSG00000197841
ENSG00000061936	ENSG00000061936
ENSG00000157429	ENSG00000143157
ENSG00000146278	ENSG00000157429
ENSG00000170260	ENSG00000146278
ENSG00000197024	ENSG00000170260
ENSG00000197128	ENSG00000197024
ENSG00000103343	ENSG00000197128
ENSG00000173480	ENSG00000173480
ENSG00000119574	ENSG00000119574
ENSG00000170265	ENSG00000170265
ENSG00000197933	ENSG00000197933
ENSG00000133250	ENSG00000133250
ENSG00000056277	ENSG00000120832
ENSG00000186280	ENSG00000186280
ENSG00000120832	ENSG00000139651
ENSG00000139651	ENSG00000136574
ENSG00000179627	ENSG00000160993
ENSG00000198205	ENSG00000147124
ENSG00000140265	ENSG00000171448
ENSG00000147124	ENSG00000197037
ENSG00000160993	ENSG00000198604
ENSG00000197037	ENSG00000070495
ENSG00000171448	ENSG00000133247
ENSG00000198604	ENSG00000235109
ENSG00000070495	ENSG00000186272

ENSG00000068024	ENSG00000170631
ENSG00000133247	ENSG00000124444
ENSG00000235109	ENSG00000178386
ENSG00000186272	ENSG00000213096
ENSG00000144802	ENSG00000196670
ENSG00000170631	ENSG00000196466
ENSG00000124444	ENSG00000141956
ENSG00000178386	ENSG00000158691
ENSG00000168062	ENSG00000197044
ENSG00000213096	ENSG00000121454
ENSG00000196670	ENSG00000187626
ENSG00000164379	ENSG00000185252
ENSG00000186260	ENSG00000197566
ENSG00000196466	ENSG00000005801
ENSG00000141956	ENSG00000101096
ENSG00000197044	ENSG00000142528
ENSG00000158691	ENSG00000188785
ENSG00000121454	ENSG00000205683
ENSG00000187626	ENSG00000185278
ENSG00000185252	ENSG00000118707
ENSG00000197566	ENSG00000081386
ENSG00000005801	ENSG00000198182
ENSG00000123411	ENSG00000131931
ENSG00000188785	ENSG00000196456
ENSG00000205683	ENSG00000112365
ENSG00000204569	ENSG00000168564
ENSG00000185278	ENSG00000161551
ENSG00000118707	ENSG00000198551
ENSG00000081386	ENSG00000124160
ENSG00000198182	ENSG00000168661
ENSG00000131931	ENSG00000184677
ENSG00000196456	ENSG00000180626
ENSG00000112365	ENSG00000153207
ENSG00000125812	ENSG00000121903
ENSG00000065526	ENSG00000249471
ENSG00000161551	ENSG00000156853
ENSG00000124160	ENSG00000139718
ENSG00000168661	ENSG00000197062
ENSG00000198551	ENSG00000102804
ENSG00000180626	ENSG00000118412
ENSG00000184677	ENSG00000198298
ENSG00000155592	ENSG00000173875
ENSG00000121903	ENSG00000215421
ENSG00000177485	ENSG00000179922
ENSG00000198081	ENSG00000181896
ENSG00000249471	ENSG00000143067
ENSG00000178764	ENSG00000143970
ENSG00000101945	ENSG00000147789
ENSG00000141026	ENSG00000198538
ENSG00000156853	ENSG00000176024
ENSG00000087152	ENSG00000186130
ENSG00000139718	ENSG00000171606
ENSG00000197062	ENSG00000187792
ENSG00000186141	ENSG00000143373
ENSG00000118412	ENSG00000115568
ENSG00000198298	ENSG00000136866
ENSG00000173875	ENSG00000267508
ENSG00000215421	ENSG00000064932
ENSG00000179922	ENSG00000254004
ENSG00000181896	ENSG00000181315
ENSG00000143067	ENSG00000183309

ENSG00000143970	ENSG00000172171
ENSG00000147789	ENSG00000143498
ENSG00000198538	ENSG00000125945
ENSG00000176024	ENSG00000152439
ENSG00000143379	ENSG00000152433
ENSG00000164916	ENSG00000175197
ENSG00000205659	ENSG00000166526
ENSG00000186130	ENSG00000125846
ENSG00000171606	ENSG00000152784
ENSG00000187792	ENSG00000083817
ENSG00000143373	ENSG00000083812
ENSG00000167685	ENSG00000116833
ENSG00000115568	ENSG00000196812
ENSG00000136866	ENSG00000196705
ENSG00000267508	ENSG00000168795
ENSG00000064932	ENSG00000152443
ENSG00000254004	ENSG00000105556
ENSG00000181315	ENSG00000180855
ENSG00000183309	ENSG00000111596
ENSG00000100201	ENSG00000196345
ENSG00000125945	ENSG00000174197
ENSG00000152439	ENSG00000083828
ENSG00000256060	ENSG00000177045
ENSG00000152433	ENSG00000155760
ENSG00000178951	ENSG00000137185
ENSG00000175197	ENSG00000196214
ENSG00000168517	ENSG00000178935
ENSG00000166526	ENSG00000172977
ENSG00000125846	ENSG00000256294
ENSG00000204304	ENSG00000180884
ENSG00000152784	ENSG00000203326
ENSG00000083817	ENSG00000179943
ENSG00000083812	ENSG00000167395
ENSG00000013619	ENSG00000169184
ENSG00000116833	ENSG00000198466
ENSG00000196812	ENSG00000186812
ENSG00000196705	ENSG00000167785
ENSG00000168795	ENSG00000079999
ENSG00000152443	ENSG00000218891
ENSG00000105556	ENSG00000213799
ENSG00000180855	ENSG00000185670
ENSG00000213024	ENSG00000204859
ENSG00000111596	ENSG00000100105
ENSG00000196345	ENSG00000118260
ENSG00000068654	ENSG00000081189
ENSG00000174197	ENSG00000213588
ENSG00000066135	ENSG00000171574
ENSG00000083828	ENSG00000177873
ENSG00000215271	ENSG00000010539
ENSG00000177045	ENSG00000148300
ENSG00000137185	ENSG00000172273
ENSG00000196214	ENSG00000169981
ENSG00000178935	ENSG00000173276
ENSG00000149308	ENSG00000078246
ENSG00000172977	ENSG00000171161
ENSG00000256294	ENSG00000196378
ENSG00000180884	ENSG00000167635
ENSG00000203326	ENSG00000185730
ENSG00000083838	ENSG00000063438
ENSG00000179943	ENSG00000197782
ENSG00000167395	ENSG00000198105

ENSG00000078900	ENSG00000196757
ENSG00000169184	ENSG00000182986
ENSG00000108175	ENSG00000124459
ENSG00000166261	ENSG00000196652
ENSG00000198466	ENSG00000166188
ENSG00000186812	ENSG00000180035
ENSG00000167785	ENSG00000186448
ENSG00000079999	ENSG00000105497
ENSG00000218891	ENSG00000186300
ENSG00000213799	ENSG00000122386
ENSG00000185670	ENSG00000140987
ENSG00000204859	ENSG00000167034
ENSG00000100105	ENSG00000167625
ENSG00000198890	ENSG00000185591
ENSG00000189298	ENSG00000197385
ENSG00000118263	ENSG00000123095
ENSG00000213588	ENSG00000112584
ENSG00000081189	ENSG00000124201
ENSG00000171574	ENSG00000111424
ENSG00000177873	ENSG00000196357
ENSG00000010539	ENSG00000123636
ENSG00000172273	ENSG00000197279
ENSG00000169981	ENSG00000189266
ENSG00000173276	ENSG00000236104
ENSG00000171161	ENSG00000214029
ENSG00000196378	ENSG00000144791
ENSG00000185730	ENSG00000106479
ENSG00000179588	ENSG00000164011
ENSG00000063438	ENSG00000167548
ENSG00000183741	ENSG00000103199
ENSG00000105866	ENSG00000127989
ENSG00000197782	ENSG00000160094
ENSG00000174282	ENSG00000102870
ENSG00000198105	ENSG00000085276
ENSG00000167528	ENSG00000085274
ENSG00000196757	ENSG00000197363
ENSG00000153922	ENSG00000197362
ENSG00000182986	ENSG00000171295
ENSG00000196652	
ENSG00000166188	
ENSG00000186448	
ENSG00000180035	
ENSG00000105497	
ENSG00000186300	
ENSG00000122386	
ENSG00000140987	
ENSG00000167034	
ENSG00000116580	
ENSG00000167625	
ENSG00000132005	
ENSG00000176182	
ENSG00000197385	
ENSG00000166886	
ENSG00000177853	
ENSG00000123095	
ENSG00000112584	
ENSG00000111424	
ENSG00000148297	
ENSG00000196357	
ENSG00000123636	
ENSG00000197279	

ENSG00000155666	
ENSG00000224470	
ENSG00000189266	
ENSG00000214029	
ENSG00000236104	
ENSG00000144791	
ENSG00000106479	
ENSG00000148411	
ENSG00000167548	
ENSG00000103199	
ENSG00000137504	
ENSG00000127528	
ENSG00000160094	
ENSG00000102870	
ENSG00000204713	
ENSG00000085276	
ENSG00000085274	
ENSG00000172530	
ENSG00000197363	
ENSG00000197362	
ENSG00000171295	

S
