# Supplementary material for: CDK7 inhibitor THZ1 inhibits MCL1 synthesis and drives cholangiocarcinoma apoptosis in combination with BCL2/BCL-XL inhibitor ABT-263
Source: Cell Death Dis. 2019 Aug 9;10(8):602. doi: 10.1038/s41419-019-1831-7 (PMC6688996; doi:10.1038/s41419-019-1831-7)
Supplement: Supplementary file 18 — Supplementary table 9. [file 41419_2019_1831_MOESM18_ESM.pdf]

GO analysis of RBE	
transcription, DNA-templated: 431 genes	regulation of transcription, DNA-templated: 347 genes
ENSG00000198521	ENSG00000146587
ENSG00000131759	ENSG00000162086
ENSG00000167981	ENSG00000198521
ENSG00000163995	ENSG00000173041
ENSG00000099381	ENSG00000167981
ENSG00000197008	ENSG00000161914
ENSG00000121413	ENSG00000099381
ENSG00000124613	ENSG00000145908
ENSG00000167840	ENSG00000197008
ENSG00000251369	ENSG00000124613
ENSG00000171425	ENSG00000167840
ENSG00000185219	ENSG00000251369
ENSG00000133740	ENSG00000171425
ENSG00000184939	ENSG00000185219
ENSG00000109787	ENSG00000168310
ENSG00000136870	ENSG00000263002
ENSG00000197857	ENSG00000160679
ENSG00000141905	ENSG00000184939
ENSG00000204366	ENSG00000109787
ENSG00000156384	ENSG00000136870
ENSG00000144026	ENSG00000189164
ENSG00000204920	ENSG00000197857
ENSG00000136367	ENSG00000204366
ENSG00000166704	ENSG00000149050
ENSG00000196092	ENSG00000144026
ENSG00000139372	ENSG00000204920
ENSG00000170608	ENSG00000136367
ENSG00000121406	ENSG00000184635
ENSG00000196152	ENSG00000104221
ENSG00000170604	ENSG00000188321
ENSG00000197016	ENSG00000188295
ENSG00000106261	ENSG00000169955
ENSG00000196150	ENSG00000178229
ENSG00000169951	ENSG00000166704
ENSG00000197937	ENSG00000196092
ENSG00000196793	ENSG00000153767
ENSG00000186496	ENSG00000110851
ENSG00000198315	ENSG00000121406
ENSG00000197841	ENSG00000196152
ENSG00000196693	ENSG00000197016
ENSG00000061936	ENSG00000170604
ENSG00000146278	ENSG00000106261
ENSG00000157429	ENSG00000198093
ENSG00000197020	ENSG00000196150
ENSG00000170260	ENSG00000169951
ENSG00000152926	ENSG00000167562
ENSG00000197024	ENSG00000196793
ENSG00000197128	ENSG00000197937
ENSG00000103343	ENSG00000198315
ENSG00000173480	ENSG00000197841
ENSG00000158805	ENSG00000196693
ENSG00000119574	ENSG00000171634
ENSG00000197935	ENSG00000061936
ENSG00000197933	ENSG00000143157
ENSG00000170265	ENSG00000157429
ENSG00000186280	ENSG00000146278
ENSG00000139651	ENSG00000197020
ENSG00000179627	ENSG00000170260
ENSG00000198205	ENSG00000152926

ENSG00000198300	ENSG00000197024
ENSG00000204644	ENSG00000133619
ENSG00000140265	ENSG00000197128
ENSG00000196275	ENSG00000173480
ENSG00000147124	ENSG00000158805
ENSG00000198604	ENSG00000119574
ENSG00000204946	ENSG00000197935
ENSG00000197037	ENSG00000197933
ENSG00000171448	ENSG00000170265
ENSG00000204947	ENSG00000171700
ENSG00000196172	ENSG00000133250
ENSG00000235109	ENSG00000186280
ENSG00000225614	ENSG00000120832
ENSG00000186272	ENSG00000187187
ENSG00000144802	ENSG00000139651
ENSG00000170631	ENSG00000204644
ENSG00000124444	ENSG00000136574
ENSG00000116668	ENSG00000177508
ENSG00000197863	ENSG00000196275
ENSG00000186376	ENSG00000147124
ENSG00000213096	ENSG00000160993
ENSG00000196670	ENSG00000198604
ENSG00000164379	ENSG00000197037
ENSG00000198807	ENSG00000171448
ENSG00000186260	ENSG00000204947
ENSG00000075407	ENSG00000070495
ENSG00000141040	ENSG00000196172
ENSG00000196466	ENSG00000133247
ENSG00000141956	ENSG00000235109
ENSG00000197044	ENSG00000225614
ENSG00000121454	ENSG00000186272
ENSG00000198429	ENSG00000170631
ENSG00000185252	ENSG00000168264
ENSG00000005801	ENSG00000124444
ENSG00000163946	ENSG00000178386
ENSG00000146243	ENSG00000147121
ENSG00000171469	ENSG00000197863
ENSG00000179111	ENSG00000213096
ENSG00000131849	ENSG00000186376
ENSG00000125798	ENSG00000196670
ENSG00000204569	ENSG00000075407
ENSG00000131848	ENSG00000196466
ENSG00000251192	ENSG00000141956
ENSG00000196199	ENSG00000197044
ENSG00000081386	ENSG00000158691
ENSG00000196458	ENSG00000198429
ENSG00000196456	ENSG00000121454
ENSG00000198551	ENSG00000187626
ENSG00000180626	ENSG00000185252
ENSG00000124664	ENSG00000197566
ENSG00000196867	ENSG00000178150
ENSG00000155592	ENSG00000005801
ENSG00000177485	ENSG00000143995
ENSG00000121903	ENSG00000163946
ENSG00000223547	ENSG00000221923
ENSG00000249471	ENSG00000146243
ENSG00000178764	ENSG00000108788
ENSG00000101945	ENSG00000171469
ENSG00000198440	ENSG00000101096
ENSG00000141026	ENSG00000161298
ENSG00000156853	ENSG00000131849

ENSG00000087152	ENSG00000142528
ENSG00000139718	ENSG00000188785
ENSG00000197062	ENSG00000205683
ENSG00000186141	ENSG00000251192
ENSG00000178665	ENSG00000185278
ENSG00000215421	ENSG00000118707
ENSG00000179922	ENSG00000081386
ENSG00000181896	ENSG00000198182
ENSG00000147789	ENSG00000131931
ENSG00000198538	ENSG00000196458
ENSG00000196437	ENSG00000196456
ENSG00000186130	ENSG00000112365
ENSG00000048052	ENSG00000161551
ENSG00000176401	ENSG00000168564
ENSG00000065029	ENSG00000134852
ENSG00000136866	ENSG00000168661
ENSG00000090612	ENSG00000124160
ENSG00000267508	ENSG00000198551
ENSG00000166135	ENSG00000180626
ENSG00000254004	ENSG00000184677
ENSG00000197497	ENSG00000245680
ENSG00000181315	ENSG00000196867
ENSG00000183309	ENSG00000153207
ENSG00000170954	ENSG00000121903
ENSG00000121297	ENSG00000198185
ENSG00000152439	ENSG00000223547
ENSG00000152433	ENSG00000249471
ENSG00000198795	ENSG00000198440
ENSG00000135164	ENSG00000174586
ENSG00000240225	ENSG00000156853
ENSG00000152784	ENSG00000139718
ENSG00000013619	ENSG00000197062
ENSG00000116833	ENSG00000118412
ENSG00000188868	ENSG00000102804
ENSG00000196705	ENSG00000198298
ENSG00000234602	ENSG00000188283
ENSG00000152443	ENSG00000173875
ENSG00000105556	ENSG00000178665
ENSG00000196417	ENSG00000237440
ENSG00000163508	ENSG00000215421
ENSG00000168395	ENSG00000179922
ENSG00000068654	ENSG00000181896
ENSG00000174197	ENSG00000143067
ENSG00000181638	ENSG00000143970
ENSG00000172977	ENSG00000103510
ENSG00000142065	ENSG00000147789
ENSG00000078900	ENSG00000198538
ENSG00000169184	ENSG00000176024
ENSG00000108175	ENSG00000196437
ENSG00000166261	ENSG00000186130
ENSG00000181450	ENSG00000171606
ENSG00000218891	ENSG00000152475
ENSG00000180787	ENSG00000143373
ENSG00000213799	ENSG00000187792
ENSG00000185670	ENSG00000172888
ENSG00000204859	ENSG00000115568
ENSG00000012504	ENSG00000176401
ENSG00000198890	ENSG00000114853
ENSG00000181666	ENSG00000136866
ENSG00000189298	ENSG00000196700
ENSG00000118263	ENSG00000267508

ENSG00000213588	ENSG00000090612
ENSG00000112561	ENSG00000077150
ENSG00000182973	ENSG00000064932
ENSG00000171574	ENSG00000254004
ENSG00000010539	ENSG00000197497
ENSG00000189079	ENSG00000181315
ENSG00000172273	ENSG00000183309
ENSG00000169981	ENSG00000170954
ENSG00000173276	ENSG00000172171
ENSG00000173275	ENSG00000143498
ENSG00000171161	ENSG00000125945
ENSG00000129173	ENSG00000152439
ENSG00000011258	ENSG00000152433
ENSG00000172819	ENSG00000175197
ENSG00000179588	ENSG00000166526
ENSG00000103460	ENSG00000198795
ENSG00000172006	ENSG00000135164
ENSG00000197782	ENSG00000125846
ENSG00000174282	ENSG00000240225
ENSG00000196757	ENSG00000083817
ENSG00000261221	ENSG00000152784
ENSG00000182986	ENSG00000083812
ENSG00000196653	ENSG00000116833
ENSG00000196652	ENSG00000196812
ENSG00000166188	ENSG00000188868
ENSG00000105497	ENSG00000196705
ENSG00000122386	ENSG00000198482
ENSG00000116580	ENSG00000185697
ENSG00000075891	ENSG00000198346
ENSG00000136944	ENSG00000168795
ENSG00000132005	ENSG00000152443
ENSG00000197385	ENSG00000105556
ENSG00000152795	ENSG00000172748
ENSG00000112584	ENSG00000180855
ENSG00000148297	ENSG00000242779
ENSG00000087095	ENSG00000196417
ENSG00000197279	ENSG00000111596
ENSG00000155666	ENSG00000196345
ENSG00000224470	ENSG00000198879
ENSG00000189266	ENSG00000174197
ENSG00000100395	ENSG00000083828
ENSG00000214029	ENSG00000256771
ENSG00000106479	ENSG00000198155
ENSG00000204611	ENSG00000138073
ENSG00000148411	ENSG00000177045
ENSG00000103199	ENSG00000181638
ENSG00000181690	ENSG00000137185
ENSG00000234284	ENSG00000130544
ENSG00000012048	ENSG00000155760
ENSG00000137504	ENSG00000256087
ENSG00000103449	ENSG00000196214
ENSG00000102870	ENSG00000178935
ENSG00000160094	ENSG00000172977
ENSG00000204713	ENSG00000142065
ENSG00000171291	ENSG00000256294
ENSG00000197363	ENSG00000203326
ENSG00000189180	ENSG00000180884
ENSG00000197362	ENSG00000127663
ENSG00000171295	ENSG00000198169
ENSG00000162086	ENSG00000179943
ENSG00000146587	ENSG00000167395

ENSG00000173041	ENSG00000169184
ENSG00000168826	ENSG00000198039
ENSG00000161914	ENSG00000198464
ENSG00000145908	ENSG00000186814
ENSG00000263002	ENSG00000198466
ENSG00000189164	ENSG00000186812
ENSG00000173894	ENSG00000181450
ENSG00000149050	ENSG00000167785
ENSG00000184635	ENSG00000079999
ENSG00000163320	ENSG00000218891
ENSG00000188321	ENSG00000180787
ENSG00000188295	ENSG00000213799
ENSG00000178229	ENSG00000185670
ENSG00000148606	ENSG00000204859
ENSG00000198093	ENSG00000100105
ENSG00000167562	ENSG00000118260
ENSG00000154222	ENSG00000181666
ENSG00000184436	ENSG00000213588
ENSG00000119866	ENSG00000081189
ENSG00000171634	ENSG00000112561
ENSG00000133250	ENSG00000182973
ENSG00000056277	ENSG00000171574
ENSG00000120832	ENSG00000177873
ENSG00000187187	ENSG00000010539
ENSG00000177508	ENSG00000189079
ENSG00000160993	ENSG00000148300
ENSG00000070495	ENSG00000172273
ENSG00000068024	ENSG00000169981
ENSG00000133247	ENSG00000078246
ENSG00000162702	ENSG00000173276
ENSG00000168264	ENSG00000171161
ENSG00000178386	ENSG00000196378
ENSG00000168062	ENSG00000173275
ENSG00000058600	ENSG00000167635
ENSG00000143458	ENSG00000011258
ENSG00000158691	ENSG00000185730
ENSG00000187626	ENSG00000063438
ENSG00000184402	ENSG00000172006
ENSG00000197566	ENSG00000197782
ENSG00000178150	ENSG00000198105
ENSG00000123411	ENSG00000256229
ENSG00000108788	ENSG00000196757
ENSG00000161298	ENSG00000186446
ENSG00000188785	ENSG00000125618
ENSG00000205683	ENSG00000182986
ENSG00000185278	ENSG00000196653
ENSG00000118707	ENSG00000124459
ENSG00000198182	ENSG00000166188
ENSG00000131931	ENSG00000196652
ENSG00000112365	ENSG00000186448
ENSG00000161551	ENSG00000180035
ENSG00000065526	ENSG00000105497
ENSG00000125812	ENSG00000167232
ENSG00000168661	ENSG00000186300
ENSG00000124160	ENSG00000122386
ENSG00000134852	ENSG00000140987
ENSG00000184677	ENSG00000167034
ENSG00000245680	ENSG00000167625
ENSG00000105662	ENSG00000075891
ENSG00000198185	ENSG00000136944
ENSG00000198081	ENSG00000185591

ENSG00000101076	ENSG00000197385
ENSG00000174586	ENSG00000123095
ENSG00000118412	ENSG00000105997
ENSG00000198298	ENSG00000167554
ENSG00000100413	ENSG00000152795
ENSG00000188283	ENSG00000112584
ENSG00000173875	ENSG00000124201
ENSG00000237440	ENSG00000111424
ENSG00000143067	ENSG00000196357
ENSG00000143970	ENSG00000087095
ENSG00000103510	ENSG00000123636
ENSG00000143379	ENSG00000149948
ENSG00000176024	ENSG00000197279
ENSG00000164916	ENSG00000196391
ENSG00000205659	ENSG00000187801
ENSG00000171606	ENSG00000089335
ENSG00000152475	ENSG00000189266
ENSG00000187792	ENSG00000100395
ENSG00000143373	ENSG00000144791
ENSG00000167685	ENSG00000214029
ENSG00000172888	ENSG00000236104
ENSG00000115568	ENSG00000106479
ENSG00000114853	ENSG00000164011
ENSG00000196700	ENSG00000204611
ENSG00000064932	ENSG00000103199
ENSG00000128908	ENSG00000167548
ENSG00000100201	ENSG00000234284
ENSG00000125945	ENSG00000128000
ENSG00000256060	ENSG00000127989
ENSG00000178951	ENSG00000136630
ENSG00000175197	ENSG00000204514
ENSG00000168517	ENSG00000196263
ENSG00000166526	ENSG00000102870
ENSG00000125846	ENSG00000160094
ENSG00000204304	ENSG00000180938
ENSG00000198146	ENSG00000085276
ENSG00000083817	ENSG00000085274
ENSG00000083812	ENSG00000171291
ENSG00000083814	ENSG00000118513
ENSG00000196812	ENSG00000197363
ENSG00000198482	ENSG00000189180
ENSG00000198346	ENSG00000197362
ENSG00000168795	ENSG00000171295
ENSG00000172748	ENSG00000187815
ENSG00000165671	
ENSG00000180855	
ENSG00000242779	
ENSG00000213024	
ENSG00000111596	
ENSG00000196345	
ENSG00000111087	
ENSG00000066135	
ENSG00000083828	
ENSG00000256771	
ENSG00000198155	
ENSG00000215271	
ENSG00000138073	
ENSG00000119608	
ENSG00000177045	
ENSG00000137185	
ENSG00000130544	

ENSG00000256087	
ENSG00000196214	
ENSG00000178935	
ENSG00000149308	
ENSG00000256294	
ENSG00000180884	
ENSG00000203326	
ENSG00000083838	
ENSG00000127663	
ENSG00000198169	
ENSG00000179943	
ENSG00000167395	
ENSG00000198039	
ENSG00000198464	
ENSG00000186814	
ENSG00000198466	
ENSG00000186812	
ENSG00000167785	
ENSG00000079999	
ENSG00000100105	
ENSG00000081189	
ENSG00000177873	
ENSG00000196378	
ENSG00000185730	
ENSG00000063438	
ENSG00000183741	
ENSG00000105866	
ENSG00000198105	
ENSG00000256229	
ENSG00000167528	
ENSG00000153922	
ENSG00000186446	
ENSG00000178919	
ENSG00000125618	
ENSG00000186448	
ENSG00000180035	
ENSG00000167232	
ENSG00000186300	
ENSG00000140987	
ENSG00000167034	
ENSG00000167625	
ENSG00000164749	
ENSG00000176182	
ENSG00000166886	
ENSG00000049246	
ENSG00000177853	
ENSG00000105997	
ENSG00000123095	
ENSG00000167554	
ENSG00000111424	
ENSG00000196357	
ENSG00000123636	
ENSG00000196391	
ENSG00000187801	
ENSG00000089335	
ENSG00000144791	
ENSG00000236104	
ENSG00000167548	
ENSG00000128000	
ENSG00000136630	
ENSG00000127528	

ENSG00000196263	
ENSG00000180938	
ENSG00000085276	
ENSG00000085274	
ENSG00000172530	
ENSG00000187815	
